# Quantitative Comparison and Chemical Profile of Different Botanical Parts of *Panax notoginseng* From Different Regions

**DOI:** 10.3389/fnut.2022.841541

**Published:** 2022-04-27

**Authors:** Mengyuan Gao, Xiunan Cao, Shujie Wei, Xuhua Huang, Huizi Ouyang, Yanxu Chang, Rui Shi, Jun He

**Affiliations:** ^1^State Key Laboratory of Component-Based Chinese Medicine, Tianjin University of Traditional Chinese Medicine, Tianjin, China; ^2^Key Laboratory for Forest Resources Conservation and Utilization in the Southwest Mountains of China, Ministry of Education, Southwest Forestry University, Kunming, China

**Keywords:** *Panax notoginseng*, chemical profile, multivariate analysis, LC-MS/MS, GC-MS

## Abstract

The root of *Panax notoginseng*, a highly valued medicine and functional food, is the main part used for medicinal purposes. However, the stems and leaves are also used in practice. To provide a chemical basis for various uses, a quantitative comparison of 18 saponins using a non-targeted metabolomics approach was established, so as to investigate the chemical profiles of the different parts of *P. notoginseng*. The established strategy revealed that roots and stems, with their similar chemical characteristics, consisted mainly of protopanaxatriol-type saponins, whereas protopanaxadiol-type saponins were principally present in the leaves. Multivariate analysis further suggested that the quality of the stems and leaves of *P. notoginseng* was significantly affected by its geographical origin. Furthermore, 52 constituents (26 non-volatile and 26 volatile) were identified as potential markers for discriminating between different parts of the plant. Taken together, the study provides comprehensive chemical evidence for the rational application and exploitation of different parts of *P. notoginseng*.

## Introduction

The root of *Panax notoginseng* (Burk.) F.H. Chen, commonly known as Sanqi or Tianqi, is a highly valuable traditional Chinese medicine, which is cultivated mainly in Yunnan and Guangxi provinces (1). Pharmacological studies have demonstrated that *P. notoginseng* exerts protective effects on the cardiovascular and cerebrovascular systems and exhibits anticarcinogenic and hepatoprotective properties (2, 3). *P. notoginseng* is more widely used as a functional food, than in medicine, because of its capability to protect the liver, regulate blood lipids, fight fatigue, and reduce hypoxic conditions. It has been reported that 47 types of healthy food containing *P. notoginseng* as an essential ingredient were approved by the State Food and Drug Administration in China (4). Phytochemical investigations have reported that *P. notoginseng* contains various constituents including saponins, amino acids, flavonoids, and polysaccharides. Among them, saponins are considered the main active components (5, 6). Since the biological activities of saponins are related to their structures, the effect of ginsenoside Rg1 with a protopanaxatriol moiety is contrary to that of ginsenoside Rb1 with protopanaxadiol as an aglycon (7, 8). Indeed, the content and type of saponins investigated are distinctly diverse in different botanical parts of *P. notoginseng* (9).

Underground parts of *P. notoginseng* are mainly used (10), although previous studies have reported that protopanaxadiol-type saponins from the stem and leaf of *P. notoginseng* had been found to possess some beneficial pharmacological effects, including antioxidative, antihyperlipidemic, and hepatoprotective activities (11–14). *P. notoginseng* stems and leaves are commonly used to treat bone fractures, calm nerves, and increase appetite (15). Owing to the different utilization of individual parts of *P. notoginseng*, differences in chemical characteristics are of great significance for authentication and ensuring the efficacy of different parts of this herb. The saponins derived from underground parts have been investigated in majority of the previous studies, while reports on aerial parts are limited (16–19). The contents of eight saponins from roots, stems, and leaves were previously compared using high-performance liquid chromatography coupled with evaporative light scattering detection (HPLC-ELSD) (8). Indeed, multiple components of an herb indicate its comprehensive efficacy, and quality assessment based on a few markers has proven to be insufficient (20). Therefore, it is necessary to determine the multiple constituents of *P. notoginseng* to assess its quality systematically and comprehensively.

Intensive research has demonstrated that geographical origin can significantly affect the quality of these herbs, as climate and environment influence biosynthesis and the accumulation of secondary metabolites in organisms (21–23). *P. notoginseng* is primarily produced in the WenShan Autonomous Prefecture, Yunnan Province, China (24). With the increasing demand for *P. notoginseng* and the obstacles faced in its continuous cropping, the production of *P. notoginseng* in the WenShan Autonomous Prefecture is insufficient; thus, cropping areas are gradually extended. However, the correlation between the quality of *P. notoginseng* and its cropping origin is not clear; therefore, it is necessary to investigate the correlation for the reasonable harvesting of this herb.

In this study, an integrated method combining quantitative comparison and a non-targeted metabolomics approach was used to verify the chemical differences between samples from different botanical parts and geographical regions. Eighteen saponins were quantitatively compared using validated ultra-high-performance liquid chromatography coupled with triple quadrupole tandem mass spectrometry (UHPLC-MS/MS) method. Additionally, multiple pattern recognition models were used to evaluate the effect of botanical parts and geographical origin on saponin content. A non-targeted metabolomics approach was subsequently applied to discriminate between the different parts of the plant. Chemical markers, including both non-volatile and volatile constituents, were identified using ultra-high-performance liquid chromatography coupled with quadrupole tandem time-of-flight mass spectrometry (UHPLC-Q-TOF-MS/MS) and gas chromatography–mass spectrometry (GC-MS) assays. This study provides comprehensive chemical evidence for the rational use of different raw materials of *P. notoginseng* in practice, thereby facilitating an overall evaluation of the relevant products.

## Materials and Methods

### Sample Collection

Twenty-five batches of the whole plant of *Panax notoginseng* were harvested in person in Yunnan Province, China, rather than being purchased from the medicinal material market. Each batch of the whole plant was dried and divided into root, stem and leaf, separately. The voucher specimens were deposited in Tianjin University of Traditional Chinese Medicine, Tianjin, China. The sample information is showed in Supplementary Table S1.

### Chemicals and Reagents

The standard compounds ginsenosides Rf, Rg1, Rg2, Re, Rd, Rb1, Rb2, Rb3, Rc, Fa, Rk1, Rg5, Rg3, and F2, as well as notoginsenoside Fe, Fd, R1, Fc (G-Rf, G-Rg1, G-Rg2, G-Re, G-Rd, G-Rb1, G-Rb2, G-Rb3, G-Rc, G-Fa, G-Rk1, G-Rg5, G-Rg3, G-F2, N-Fe, N-Fd, N-R1, and N-Fc), with purity > 98%, were purchased from Chengdu DeSiTe Biological Technology Co., Ltd (Chengdu, China). The structures of saponins are shown in Supplementary Figure S1. Methanol and acetonitrile of chromatographic grade were purchased from Fisher company (Thermo Fisher Scientific Co. Ltd. Shanghai, China). Formic acid was obtained from the ROE. The distilled water was purified using a Milli-Q system (Millipore, Bedford, MA, USA).

### Preparation of Standard and Sample Solution

Stock solutions of G-Rf, G-Rg1, G-Rg2, G-Re, G-Rd, G-Rb1, G-Rb2, G-Rb3, G-Rc, G-Fa, G-Rk1, G-Rg5, G-Rg3, G-F2, N-Fe, N-Fd, N-R1, and N-Fc were dissolved in methanol and serially diluted to plot the standard curves.

All dried samples were grounded and the powder was passed through a sieve (hole diameter, 0.45 mm). Powdered samples (20 mg) were ultrasonically extracted in 20 mL of methanol for 40 min. After cooling, the resulting mixture was centrifuged, the supernatant was filtered through 0.22 μm nylon membranes, and the obtained solutions were stored at 4°C for LC-MS/MS analysis.

Powdered samples (1 g) were sonicated in 50 mL of N-hexane for 40 min and filtered through a 0.22 μm nylon membrane to obtain the sample solutions for GC-MS analysis. All the solutions were stored at 4°C prior to analysis.

### UHPLC-MS/MS Analysis

An Agilent 1290 UHPLC system (Agilent Technologies Inc., Palo Alto, CA, USA) coupled to an Agilent 6470 triple quadrupole tandem mass spectrometer with electrospray ionization (ESI) source was used for the quantitative analysis. An ACQUITY UPLC BEH C18 column (2.1 ×100 mm, 1.7 μm; Waters, Milford, MA, USA) was used for chromatographic separation, and the column temperature was maintained at 25°C. The binary gradient elution system consisted of 0.1% formic acid (solvent A) and acetonitrile (solvent B). The solvent gradient was as follows: 0–1 min, 25–33% B; 1–5 min, 33–33% B; 5–7 min, 33–41% B; 7–9 min, 41–41% B; 9–10 min, 41–59% B; 10–15 min, 59–59% B. The flow rate was 0.3 mL/min and the injection volume was 5 μL.

The mass spectrometer was operated in negative mode. The optimized mass conditions were as follows: gas temperature, 300°C; gas flow rate, 7 L/min; nebulizer, 35 psi; sheath gas temperature, 250°C; sheath gas flow, 12 L/min; capillary voltage, 4,000 V. Multiple reaction monitoring (MRM) mode was applied for the quantitative analysis of different compounds. An MRM diagram is shown in Supplementary Figure S2. The optimal mass spectral parameters and ion patterns are presented in Supplementary Table S2.

### UHPLC-Q-TOF-MS/MS Analysis

The non-targeted metabolomics study was performed using an Agilent 1290 Infinity UHPLC system equipped with an Agilent 6520 Q-TOF instrument. A Waters UPLC®BEH C18 column (2.1 ×100 mm, 1.7 μm, Waters, Milford, MA, USA) was used for chromatographic separation at a temperature of 40°C. The mobile phase consisted of 0.1% formic acid in water (A) and acetonitrile (B) at a flow rate of 0.3 mL/min. The solvent gradient was set as follows: 0–5 min, 5–15% B; 5–11 min, 15–30% B; 11–25 min, 30–38% B; 25–30 min, 38–90% B; 30–38 min, 90–90% B. The injection volume for each sample was 5 μL.

The mass spectrometer was operated in both positive and negative modes with a scanning range of *m/z* 50–1,500 and a scanning rate of 1 spectra/s. High resolution (4 GHz, High Res Mode) was used. The optimized instrumental parameters were as follows: capillary temperature, 350°C; drying gas (N_2_) flow rate, 11 L/min; nebulizer pressure, 40 psi; collision energy, 45 V; fragmentor voltage, 135 V.

### GC-MS Analysis

The volatile components were analyzed using a Shimadzu QP 2010 GC-MS system equipped with an AOC-20i Autosampler. Chromatographic separation was performed on a DB-5 MS column (0.25 μm ×0.25 mm ×30 m). The temperature program was set as follows: 40°C for 0 min, 40–190°C in increments of 7°C/min, 190–230°C in increments of 2°C/min, and 230°C for 1 min.

Mass spectrometry was performed in electron impact (EI) mode and full scan mode at *m/z* 50–1,000. The temperatures of ion source and interface were 230 and 250°C, respectively. The detector voltage was 1.3 kV.

### Method Validation

A calibration curve for each saponin was constructed using a linear regression model, and linearity was verified using correlation coefficients (r^2^). The lower limits of detection (LLODs) and lower limits of quantification (LLOQs) were estimated as the minimum concentration giving signal-to-noise ratios (S/N) of 3 and 10, respectively. Instrument precision was determined by intra- and interday variations. Relative standard deviation (RSD) was calculated as a measure of precision. Repeatability was evaluated by performing six replicate analyses on the same sample. In the stability test, the sample solutions were stored at room temperature and then analyzed by replicate injections at 0, 2, 4, 8, 12, and 24 h. Recovery was carried out by spiking a sample with the mixed standards, and the recovery rate was calculated using the following formula: recovery rate (%) = (observed amount – original amount)/spiked amount ×100%.

The UHPLC-Q-TOF-MS/MS and GC-MS method were validated in terms of precision, repeatability, and stability. Sample batch 10 from WenShan Autonomous Prefecture was randomly selected as quality control sample for the methodological investigation. The precision was evaluated by observing the intraday variations of sample for six consecutive times. The repeatability was accessed by preparing six replicate samples. The stability was obtained by detecting one sample at 0, 6, 12, 18, and 24 h. Fifteen chromatographic peaks were randomly selected to calculate the RSDs of peak area and retention time (RT) in order to investigate precision, repeatability, and stability.

### Multivariate Analysis

The UHPLC-Q-TOF-MS/MS and GC-MS data of different botanical parts of *P. notoginseng* from different geographical regions were exported in MZ format using the Agilent Masshunter analysis software and GC-MS Postrun Analysis software, respectively. The peak finding, alignment, and filtering of the raw data were preprocessed using R software to obtain the retention time (Rt), mass-to-charge ratio (m/z), and peak strength of each compound. Finally, the obtained data was imported into Simca-P 14.1 (Umetrics, Umea, Sweden) for multivariate statistical analysis, including hierarchical cluster analysis (HCA), principal component analysis (PCA) and orthogonal partial least squares discriminant analysis (OPLS-DA). Potential chemical markers to differentiate the parts of *P. notoginseng* were screened according to the variable importance in the projection (VIP) value. R2 (cum) and Q2 (cum) values were used to validate the model. R2 implied the explanation capability toward original data and Q2 indicated the prediction ability of the model.

## Results and Discussions

### Method Validation

The correlation coefficient values (*r*^2^ > 0.9991) indicated good correlation between the concentrations of the investigated compounds and their peak areas within the respective test ranges. The calibration results are summarized in Supplementary Table S3. Precision was determined by the relative standard deviations (RSDs <3.1%) for the 18 investigated components, indicating the acceptable precision of the developed method. In repeatability testing, the RSDs were in the range of 0.8–4.7%, demonstrating that the developed method had satisfactory repeatability. As for stability, the RSDs of the peak areas of the 18 saponins were lower than 5.2%, indicating that it was feasible to analyze samples within 24 h. The accuracy was validated by the recovery rate, and the results showed that the mean recovery rates of the 18 saponins were in the range of 86.1–108.4%, with RSDs <5.9%.

The RSDs of retention time and peak area of precision, repeatability, and stability were <0.3% and 8.7% using UHPLC-Q-TOF-MS/MS and GC-MS analysis, which validated that the established method was precise for differential component analysis of different parts of *P. notoginseng*.

### Comparative Analysis of *P. notoginseng* From Different Geographical Regions

**Figure 1:**
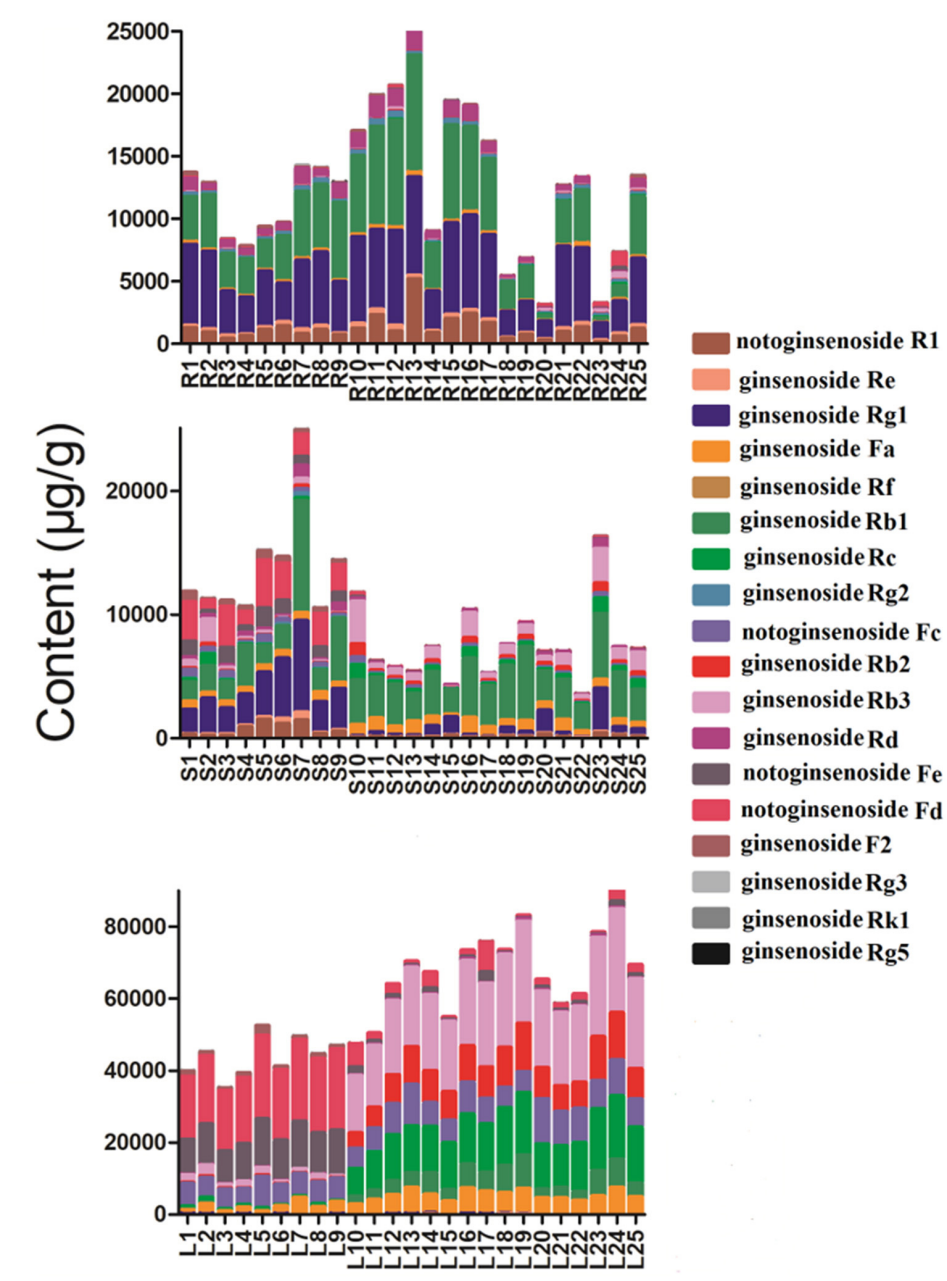
The total contents of 18 saponins in different parts of *P. notoginseng* from different batches (R, Root; S, Stem; L, Leaf).

**Figure 2:**
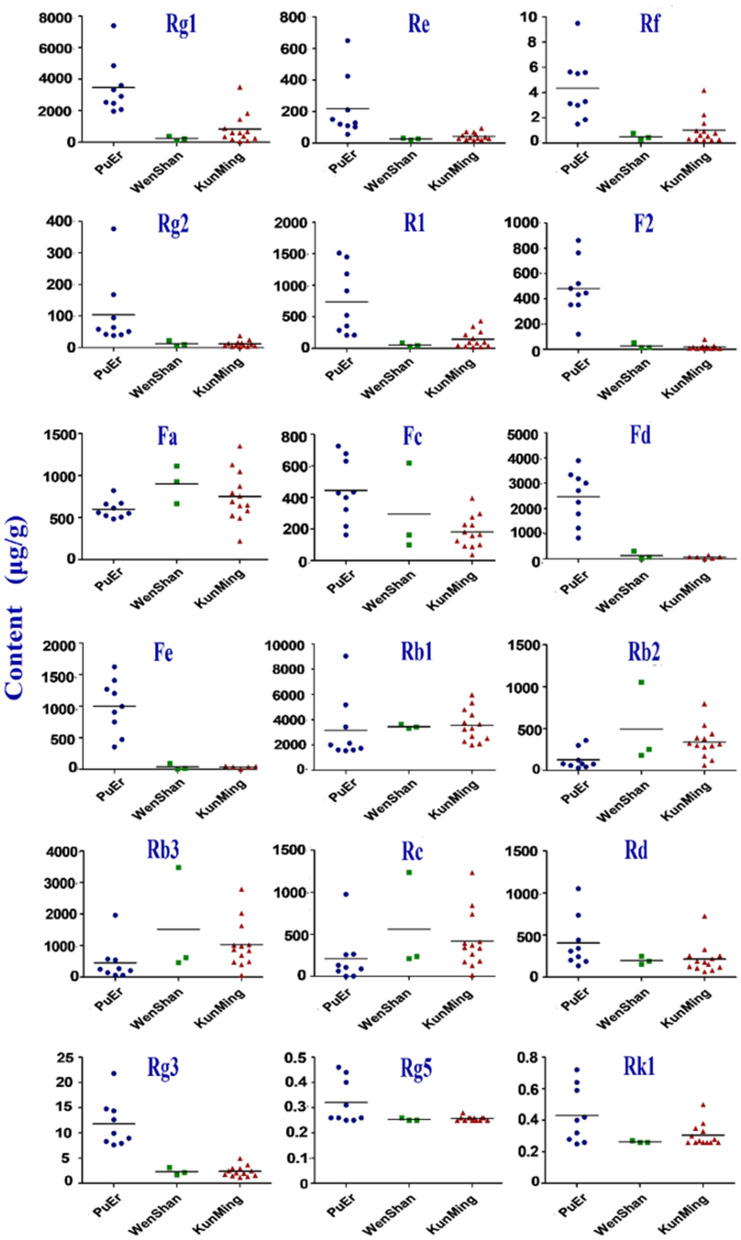
Contents of 18 saponins in stem of *P. notoginseng* from different origins.

**Figure 3:**
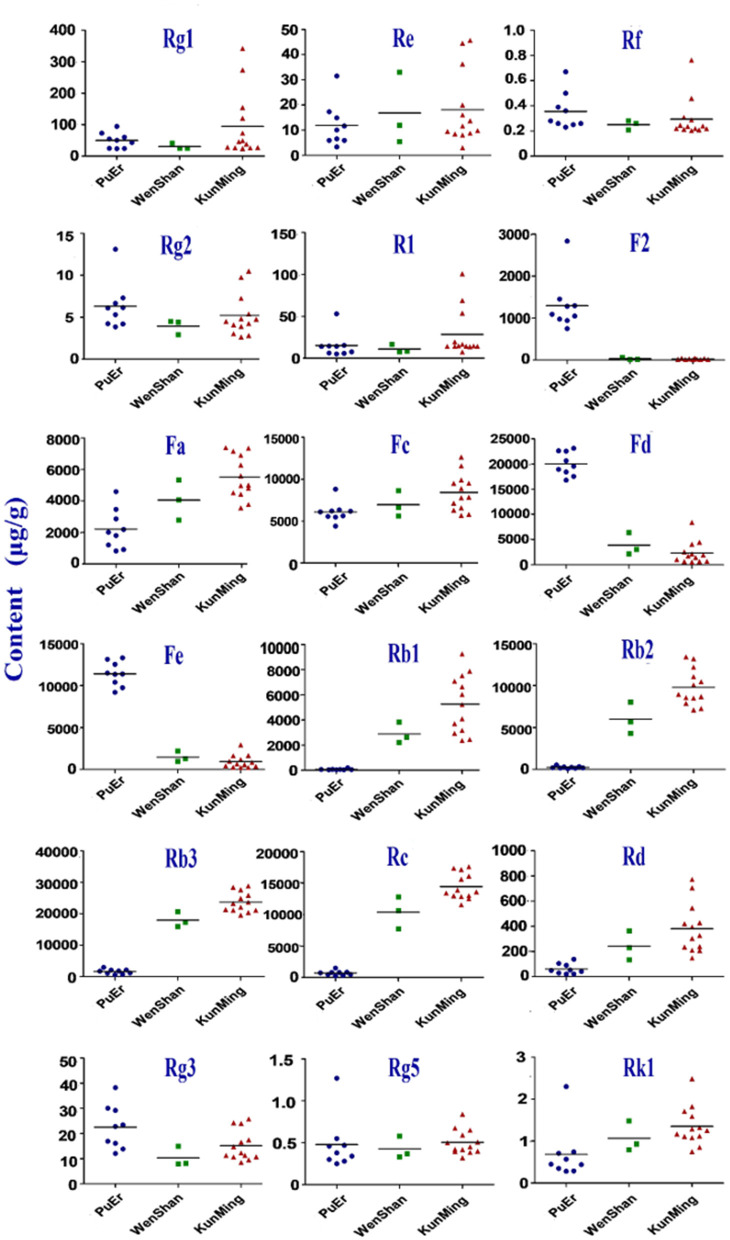
Contents of 18 saponins in leaf of *P. notoginseng* from different origins.

Geographical regions are usually deemed to affect biosynthesis and accumulation of secondary metabolites in organisms, thereby contributing to various qualities of traditional Chinese medicine (25, 26). *P. notoginseng* is produced mainly in Yunnan Province, China, accounting for more than 90% of the worldwide production. In this study, roots, stems, and leaves of *P. notoginseng* were collected from PuEr city, WenShan Autonomous Prefecture, and KunMing city of Yunnan province to analyze the influence of geographical regions on the quality of *P. notoginseng*. The contents of the 18 saponins are presented in Supplementary Table S4, which varied greatly among different batches of samples. As shown in Figure 1, the higher content of G-Rg1 and G-Rb1 in the stems of batch 7 led to a significantly higher subtotal saponin content. Specifically, the contents of N-Fd, G-Rb3, and G-Rc in the leaves were significantly different among batches 1–9 (PuEr city) when compared to the others. Individually, with the exception of G-Fa, G-Rb1, G-Rb2, G-Rb3, and G-Rc, which are present in the stems from PuEr city, they were significantly higher than those from WenShan Autonomous Prefecture and KunMing city, whereas G-Rf, G-Rg2, G-F2, N-Fd, N-Fe, and G-Rg3 in leaves from WenShan Autonomous Prefecture and KunMing city were less abundant than those from PuEr city (Figures 2, 3).

PCA is an unsupervised pattern recognition method that is often used to sort unclassified samples into groups, and it reduces the dimensionality of the original dataset by explaining the correlation among a large number of variables in terms of a smaller number of underlying factors without losing much information (27, 28). The OPLS-DA model can filter out random noise and eliminate the effects of irrelevant metabolite variability to improve the effectiveness and analytical capability of the model. PCA and OPLS-DA plots were generated to analyze the effect of geographical regions on the content of saponins from different parts of *P. notoginseng* (Supplementary Figure S3). The results based on the analyses from the roots showed that the data from these three cities superimposed on each other [Supplementary Figure S3 (1)], indicating that there were some differences in the chemical profiles of the roots derived from these three cities, but they were not completely differentiated according to their origin. In addition, the stem and leaf results presented the same classification pattern. Samples from PuEr city were in the first category, while other samples from WenShan Autonomous Prefecture and KunMing city belonged to the second category. The two categories shown in the PCA and OPLS-DA score plots were separated from each other. Geographically, WenShan Autonomous Prefecture and KunMing city are located in the middle eastern part of Yunnan Province, while PuEr city is located in the west, demonstrating that geographical location has a significant impact on the quality of the stems and leaves of *P. notoginseng*. Furthermore, consistent with the quantitative results shown in Figure 1, data from the analyses on stems from PuEr City were clustered in a relatively wider region [Supplementary Figure S3 (2)], indicating that the stem qualities were not stable. All results of the multivariate statistical analysis were consistent with those of the quantitative analysis.

### Comparative Analysis of Different Parts of *P. notoginseng*

Previous studies had elucidated that the type and content of saponins varied extremely in different parts of *P. notoginseng* (29–31). To investigate the variation of the chemical profiles among different botanical parts of *P. notoginseng*, we collected the roots, stems and leaves for the comparative study.

**Figure 4:**
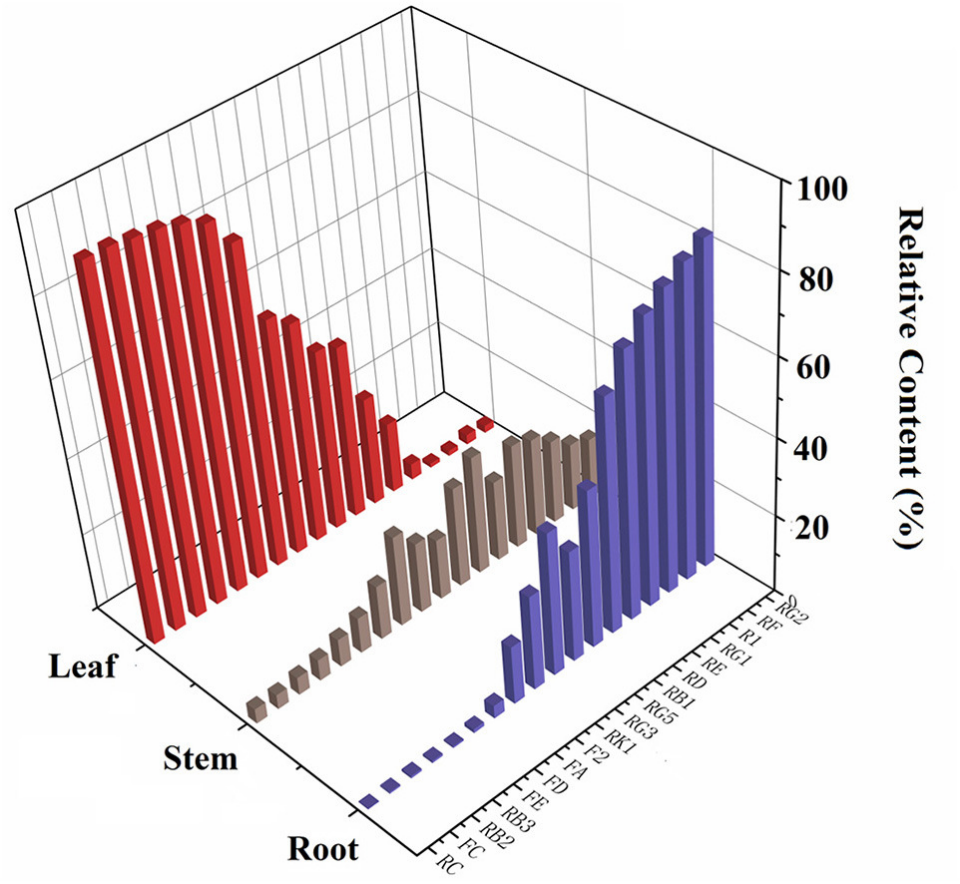
The relative content of 18 saponins in different parts of *P. notoginseng* (Relative content = individual part content/sum contents of all parts).

The validated method was used to determine 18 saponins in the roots, stems, and leaves of *P. notoginseng*. The contents of 18 saponins are listed in Supplementary Table S4. The results revealed that there were significant variations among individual parts of *P. notoginseng* in terms of both the average content and relative composition of the total amount of saponins. The contents of N-Rg1 and N-Rb1 in the roots appeared to be higher than those in the stems and leaves. G-Rb1 was present in the highest quantity in the stem, while the content of G-Rg1 varied greatly among different batches. Additionally, the amount of G-Rb3, G-Rc, and N-Fd in the leaves were much higher than those in the roots and stems. As illustrated in Figure 1, the total content of the 18 saponins was significantly different in the different botanical parts of *P. notoginseng*. The total amount of saponins in the leaves was almost 4–8 fold higher than the roots and stems. Individually, the higher content of G-Rb3 and G-Rc in the leaves led to a significantly higher content of subtotal saponins. Moreover, the relative content of the investigated saponins was used to determine the type of saponins in the different parts of the plant. As shown in Figure 4, ginsenosides Rc and Rg2 were the most abundant ones in leaves and roots, respectively, with the greatest variation among individual parts in the relative composition of the total amount of saponins. The content of protopanaxadiol (PPD)-type saponins (G-Rb1, G-Rb2, G-Rb3, G-Rg3, G-Rc, G-Rd, G-F2, N-Fc, G-Fa, N-Fe, and N-Fd) in leaves was higher than the root and stem, whereas the protopanaxatriol (PPT)-type saponins (G-Rg1, G-Re, G-Rf, G-Rg2, and N-R1) showed the opposite trend.

The PCA plot was first adopted for exploratory analysis of differences among the roots, stems, and leaves of *P. notoginseng*. The first component of the sample demonstrated 53.8% of the systematic variation. As displayed in Supplementary Figure S4A, the leaf samples tended to cluster to the left, whereas the roots and stems were scattered on the right. In OPLS-DA scores plot (Supplementary Figure S4B), the root was separated from the stem, but was still very close to it. The results indicated an overall similar chemical profile of roots and stems, although they were extremely different from the leaves, which was consistent with the results obtained from quantitative analysis.

### Identification of Chemical Markers

Untargeted metabolomics has been widely used to compare the overall metabolic composition between samples, owing to its ability to profile diverse classes of metabolites (32). It's a systematic method that can process and evaluate varieties of metabolite data, and has great value in plant phenotyping analysis (33). Nowadays, it has been extended to various research fields, such as biomarkers discovery and the quality assessment of CHMs (34). Chromatography and spectrometry are the most commonly used metabolite analysis methods because of their high separation capacity and sensitivity (35). The UHPLC-Q-TOF-MS/MS method has been widely utilized for profiling metabolites because of its high-resolution mass, precision, and sensitivity. In the present study, based on the chemical analysis of the roots, stems, and leaves using the UHPLC-Q-TOF-MS/MS method (Tables 1, 2), indicated that the type of components varied in the different botanical parts, with some saponins present in specific parts. The collected data was further exported into Simca-P 14.1 for hierarchical cluster analysis (HCA), the results showed that the roots, stems, and leaves of *P. notoginseng* were divided into three clusters (Supplementary Figure S5). In order to further screen the chemical markers between roots, stems and leaves, OPLS-DA model was built using the data matrix of three medicinal parts of *P. notoginseng*. The R2Y (cum) = 0.984 and Q2 (cum) = 0.972 indicated a good explanation and prediction ability of this model. As shown in Supplementary Figure S6, it could lead to better grouping results for separation of the roots, stems, and leaves. The data showed that the roots and stems were very close in space, consistent with the results obtained from the multivariate analysis of quantification. Furthermore, the results verified the high similarity between the chemical characteristics of the roots and stems. To further investigate the importance of each variable in distinguishing different parts of *P. notoginseng*, compounds with VIP values >1 are summarized in Supplementary Table S5 (36). Twenty-six chemical markers, including amino acids and saponins, were identified to differentiate the roots, stems, and leaves of the plant based on the accurate mass, retention time, and targeted MS/MS data. Besides, the ROC curve was generated to summarize the classification ability of this model. As shown in Supplementary Figure S7, the ROC curve passed through the upper left corner and AUC (area under the ROC curve) was 1, indicating that the 26 markers had a perfect discrimination ability which is more helpful to determine the molecular weight and formula of compounds. An example of the potential markers identified using MS/MS data is shown in Supplementary Figure S8, which exhibits a fragmentation pattern corresponding to the loss of the glycosidic units.

**Table 1:** Possible non-volatile chemicals in root, stem and leaf of *P. notoginseng* under the mode of positive ions.

**No**.	**Retention time (min)**	**m/z**	**Fragment ion**	**Loading form**	**Compound**	**Molecular formula**	**Root**	**Stem**	**Leaf**
1	0.92	175.1182	156.0748, 133.0634	[M+H]^+^	l-arginine	C_6_H_14_N_4_O_2_	+	+	+
2	1.54	132.1011	69.0694	[M+H]^+^	l-isoleucine	C_6_H_13_NO_2_	+	+	+
3	6.11	649.1459	303.0539	[M+Na]^+^	quercetin-3-o-sophorose	C_27_H_30_O_17_	-	+	+
4	11.41	925.4491	441.3714, 423.3611, 405.3503	[M+Na]^+^	chikusetsusaponin L5	C_46_H_78_O_17_	+	+	-
5	16.44	793.4678	441.3707, 423.3606	[M+Na]^+^	ginsenoside F5	C_41_H_70_O_13_	+	+	+
6	17.79	527.3775	365.1063	[M+Na]^+^	notoginsenic acid β-sophoroside	C_22_H_32_O_13_	+	+	+
7	29.81	627.3334	447.4221	[M+Na]^+^	ginsenoside Rh3/isomer	C_36_H_60_O_7_	+	-	-

*-, not detected; +, detected*.

**Table 2:** Possible non-volatile chemicals in root, stem and leaf of *P. notoginseng* under the mode of negative ions.

**No**.	**Retention time (min)**	**m/z**	**Fragment ion**	**Loading form**	**Compound**	**Molecular formula**	**Root**	**Stem**	**Leaf**
1	1.07	387.1137	1007.3659, 341.1084	[M+COOH]^−^	sucrose	C_12_H_22_O_11_	+	+	+
2	7.18	861.4813	851.4506	[M+COOH]^−^	notoginsenoside M	C_42_H_72_O_15_	+	+	-
3	10.34	979.5365	817.3952, 799.4703	[M-H]^−^	notoginsenoside E	C_48_H_82_O_20_	+	+	-
4	10.46	977.5289	931.5225, 799.4802, 637.4292, 475.3767	[M+COOH]^−^	n-Notoginsenoside R1	C_47_H_80_O_18_	+	+	+
5	10.94	845.4883	799.4805, 637.4282, 475.3764	[M+COOH]^−^	ginsenoside Rg1	C_42_H_72_O_14_	+	+	+
6	10.96	991.5432	945.5362, 799.4810, 637.2863, 475.3768	[M+COOH]^−^	ginsenoside Re	C_42_H_82_O_18_	+	+	+
7	11.47	885.4825	841.4940	[M-H]^−^	malonyl-ginsenoside Rg1/isomer	C_45_H_74_O_17_	+	+	+
8	12.08	961.5212	799.4658, 637.2995, 475.1625	[M-H]^−^	notoginsenoside R3/R6/N/20-o-glucoginsenoside Rf	C_48_H_82_O_19_	+	-	-
9	12.11	1005.5220	959.5172, 797.4677, 635.4146, 473.3646	[M+COOH]^−^	notoginsenoside G/isomer	C_48_H_80_O_19_	+	+	-
10	12.34	1167.5718	1157.5445, 1121.5667	[M+COOH]^−^	notoginsenoside B	C_54_H_90_O_24_	+	+	-
11	12.42	1139.5774	1093.5730, 781.4690	[M+COOH]^−^	floranotoginsenoside A/D	C_53_H_90_O_23_	-	-	+
12	12.56	1155.5701	1109.5681, 797.3674, 635.3066	[M+COOH]^−^	floranotoginsenoside B/C	C_53_H_90_O_24_	-	-	+
13	12.79	1139.5770	1093.5763, 637.4125, 619.2562, 475.3720	[M+COOH]^−^	gypenoside LXIX	C_53_H_90_O_23_	-	-	+
14	13.43	1137.5647	1091.5593, 779.4295	[M+COOH]^−^	yesanchinoside G/isomer	C_53_H_88_O_23_	-	-	+
15	13.48	1371.6727	1239.6115, 1107.6055, 945.5295, 783.4825, 621.4325, 459.3796	[M-H]^−^	notoginsenoside D/T	C_64_H_108_O_31_	+	+	+
16	14.38	1239.6320	945.5376, 783.4803, 621.4244, 459.3769	[M-H]^−^	ginsenoside Ra3	C_59_H_100_O_27_	+	-	-
17	14.63	845.4863	799.4760, 637.4247, 475.3752	[M+COOH]^−^	ginsenoside Rf	C_42_H_72_O_14_	+	+	+
18	14.81	1269.6467	1305.6202, 1107.5857, 945.5341, 783.4812, 621.4331, 459.3794	[M-H]^−^	ginsenoside Ra0	C_60_H_102_O_28_	+	+	+
19	15.13	783.4825	637.2995, 619.3109	[M-H]^−^	ginsenoside Rg2	C_42_H_72_O_13_	+	+	+
20	15.17	1239.6344	945.5355, 783.4825, 621.4348, 459.3800	[M-H]^−^	notoginsenoside Fa	C_59_H_100_O_27_	+	+	+
21	15.23	815.4758	805.4471, 769.4710	[M+COOH]^−^	notoginsenoside R2/isomer	C_41_H_70_O_13_	+	+	-
22	15.31	1093.6056	637.4277, 475.3758	[M-H]^−^	yesanchinoside H	C_53_H_90_O_23_	+	-	-
23	15.97	1209.6240	945.5345, 783.4848, 603.4214	[M-H]^−^	ginsenoside Ra1/Ra2	C_58_H_98_O_26_	+	-	-
24	16.31	1107.5921	783.4887, 621.4337, 459.3793	[M-H]^−^	ginsenoside Rb1	C_54_H_92_O_23_	+	+	+
25	16.36	1239.6320	1107.5920, 945.5385, 783.4858, 621.4334	[M-H]^−^	notoginsenoside R4	C_59_H_100_O_27_	+	+	-
26	16.84	1193.5932	1149.6019	[M-H]^−^	malonyl-ginsenoside Rb1/isomer	C_57_H_94_O_26_	+	+	+
27	16.92	1427.6578	1341.6622, 1209.6238	[M-H]^−^	malonyl-notoginsenoside Q	C_66_H_108_O_33_	-	-	+
28	17.27	1077.5810	945.5368, 783.4848, 621.4334, 459.3796	[M+COOH]^−^	ginsenoside Rc	C_53_H_90_O_22_	+	+	+
29	17.42	1209.6226	1077.5786, 945.5382, 783.4844, 621.4332, 459.3799	[M-H]^−^	notoginsenoside Fc	C_58_H_98_O_26_	+	+	+
30	18.45	1077.5810	945.5372, 783.4855, 621.4338, 459.3796	[M-H]^−^	ginsenoside Rb2	C_53_H_90_O_22_	+	+	+
31	18.81	1077.5814	783.4665, 621.4338, 459.3812	[M-H]^−^	ginsenoside Rb3	C_53_H_90_O_22_	+	+	+
32	19.17	683.4337	673.4026, 637.4305, 475.3785	[M+COOH]^−^	ginsenoside Rh1	C_36_H_62_O_9_	-	+	+
33	19.48	1077.5800	945.5374, 915.5171, 783.4874, 621.4293	[M-H]^−^	notoginsenoside L	C_53_H_90_O_22_	+	+	+
34	21.01	991.5453	945.5372, 783.4864, 621.4324, 459.3804, 375.2823	[M+COOH]^−^	ginsenoside Rd	C_48_H_82_O_18_	+	+	+
35	21.13	1163.5800	1077.5764, 945.5368, 621.4345, 459.3765	[M-H]^−^	malonyl-ginsenoside Rc	C_56_H_92_O_25_	-	-	+
36	21.28	991.5445	945.5372, 621.4338	[M+COOH]^−^	notoginsenoside K	C_48_H_82_O_18_	+	+	+
37	21.33	1123.5844	1077.5794, 945.5334, 783.4638	[M+COOH]^−^	vinaginsenoside R7	C_53_H_90_O_22_	-	-	+
38	21.68	1031.5377	987.5473	[M-H]^−^	malonyl-ginsenoside Rd/isomer	C_51_H_84_O_21_	+	+	+
39	22.21	987.5476	945.5352, 783.4858, 621.4326, 459.3826	[M-H]^−^	pseudoginsenoside Rc1	C_50_H_84_O_19_	+	+	+
40	23.36	991.5442	981.5115, 945.5362, 783.4858, 621.4307, 459.3774	[M+COOH]^−^	gypenoside XVII	C_48_H_82_O_18_	+	+	+
41	23.83	769.4714	637.4266, 475.3758	[M-H]^−^	ginsenoside F3/notoginsenoside R2	C_41_H_70_O_13_	+	+	+
42	24.11	1047.5696	961.5280, 637.4269	[M-H]^−^	malonyl-vinaginsenoside R4	C_51_H_84_O_22_	-	+	+
43	25.04	961.5355	915.5261, 621.4340, 459.3814	[M+COOH]^−^	notoginsenoside Fe	C_47_H_80_O_17_	+	+	+
44	26.65	961.5353	915.5269, 783.4862, 621.4337, 459.3848	[M+COOH]^−^	notoginsenoside Fd	C_47_H_80_O_17_	+	+	+
45	26.93	1093.5710	1047.5681, 915.5233, 783.4735, 621.4302, 459.3735	[M+COOH]^−^	notoginsenoside P/O	C_52_H_88_O_21_	-	+	+
46	27.33	1001.5233	915.5290, 783.4888	[M-H]^−^	malonyl-notoginsenoside fe/malonyl-vinaginsenoside R18	C_50_H_82_O_20_	+	+	+
47	27.65	829.4917	783.4900, 621.4327, 459.3799	[M+COOH]^−^	ginsenoside F2	C_42_H_72_O_13_	+	+	+
48	28.12	955.4846	793.4340	[M-H]^−^	ginsenoside Ro	C_48_H_76_O_19_	+	+	-
49	28.26	829.4930	783.4882, 459.2023	[M+COOH]^−^	ginsenoside Rg3	C_42_H_72_O_13_	+	+	+
50	29.59	667.4374	621.3441, 459.3856	[M+COOH]^−^	ginsenoside Rh2	C_36_H_62_O_8_	+	+	-
51	29.64	811.4825	765.4769, 603.4232	[M+COOH]^−^	ginsenoside Rg5/Rk1	C_42_H_70_O_12_	+	+	+
52	32.59	637.4272	475.1973	[M-H]^−^	ginsenoside F1	C_36_H_62_O_9_	-	+	+

*-, not detected; +, detected*.

**Figure 5:**
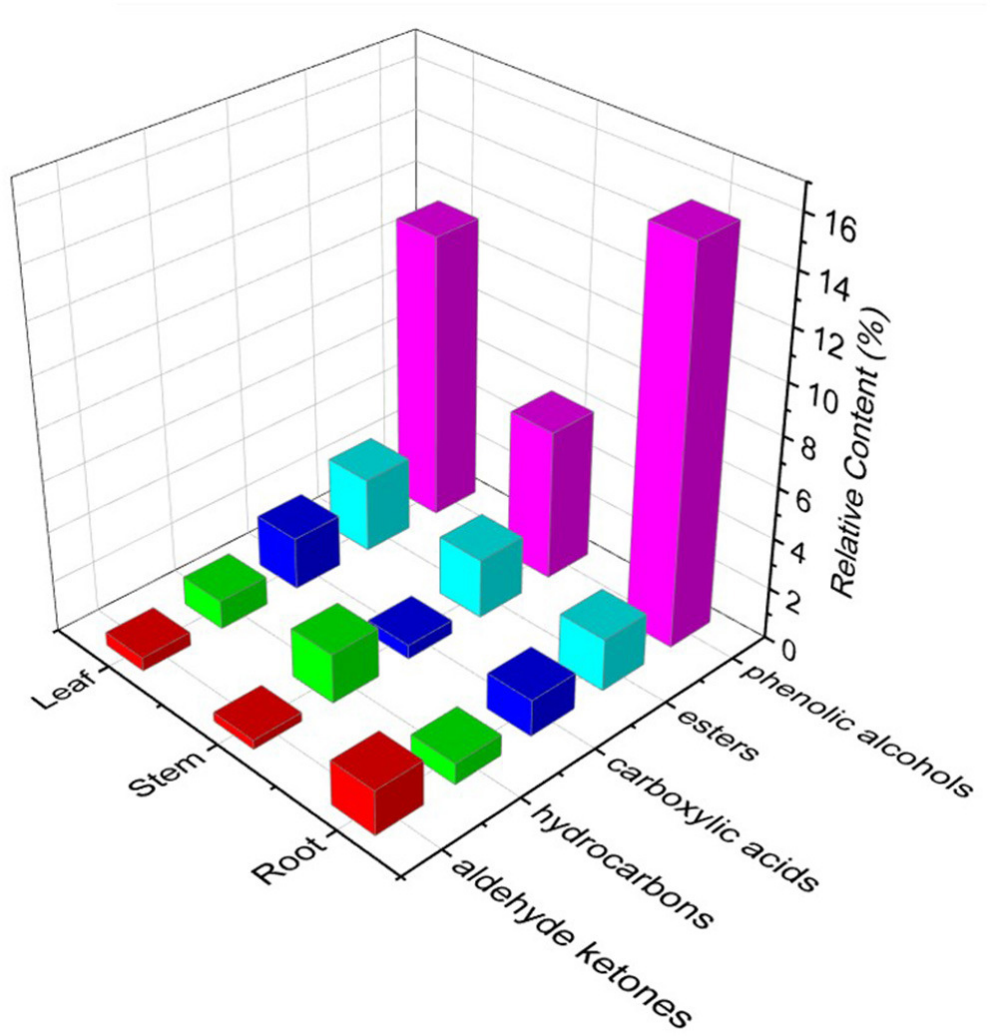
Relative content of the volatiles in root, stem and leaf of *P. notoginseng*.

Volatile constituents in plant materials are considered to possess insecticidal, biological signaling, and antifungal properties, which are of significant interest to the food and pharmaceutical industries (37). In the present study, GC-MS was performed to analyze the volatile constituents of different parts of *P. notoginseng*, and an untargeted metabolomics approach was subsequently applied to investigate the volatile markers responsible for the differentiation of roots, stems, and leaves of the plant. The possible volatile constituents were identified by comparing the mass spectral fragmentation patterns with those stored in the NIST 08 and NIST 08s spectral libraries, and the relative amounts of the identified compounds were obtained based on the percentage of the relative peak area. As summarized in Table 3, 57 volatile constituents were identified, and the compositions presented considerable chemical polymorphism among different parts of *P. notoginseng*. There were 15 compounds shared among the volatiles in the roots, stems, and leaves, including alkenes, alkanes, carboxylic acids, esters, and alcohols. Falcarinol, the dominant active compound among the identified constituents, has been reported to exhibit anticancer, sedative, and hypotensive effects (37). The falcarinol content was maximum in the stems of *P. notoginseng*, suggesting that this individual part can be utilized in a better way. The identified volatiles mainly consisted of hydrocarbons, phenolic alcohols, aldehyde ketones, carboxylic acids, and esters; their relative contents are shown in Figure 5. Phenolic alcohols were the most abundant volatile constituents in the three parts of *P. notoginseng*. The content of aldehyde ketones, hydrocarbons, and carboxylic acids varied greatly among the different botanical parts, while no significant difference was observed in the ester content. Multivariate statistical analysis was applied to further investigate the differences in the volatile constituents of the different parts of *P. notoginseng*. The profile of volatiles in the roots, stems, and leaves was visualized using an OPLS-DA model. Clear differentiation of roots, stems, and leaves is observed in Supplementary Figure S9, indicating that there were significant differences in the volatile constituents of the different parts of *P. notoginseng*. The importance of each variable for distinguishing different parts of the plant was subsequently inspected to screen out volatile chemical markers with VIP > 1 (Supplementary Table S6), all of which played a significant role in the differentiation of the roots, stems, and leaves of *P. notoginseng*.

**Table 3:** Volatile constituents in root, stem and leaf of *P. notoginseng*.

**No**.	**Compound**	**Molecular formula**	**CAS**	**m/z**	**Similarity**			**Relative content %**		
					**Root**	**Stem**	**Leaf**	**Root**	**Stem**	**Leaf**
1	4-methylhept-1-ene	C_8_H_16_	131351-05-8	112.21	88	93	93	0.01	0.04	0.02
2	2,4-dimethyl-1-heptene	C_9_H_18_	19549-87-2	126.24	97	98	98	0.62	1.8	1.01
3	2-ethyldodecan-1-ol	C_14_H_30_O	19780-33-7	214.39	90	89	82	0.08	0.02	0.02
4	2-hexyl-1-decanol	C_16_H_34_O	2425-77-6	242.44	87	87	87	0.81	1.06	0.89
5	11-methyldodecan-1-ol	C_13_H_28_O	27458-92-0	200.36	89	83	89	1.31	0.02	1.67
6	8-methylnonyl methacrylate	C_14_H_26_O_2_	29964-84-9	226.36	89	89	89	0.54	1.2	0.86
7	(5-methyl-2-propan-2-ylhexyl) acetate	C_12_H_24_O_2_	40853-55-2	200.32	81	85	86	0.01	0.01	0.02
8	butyl 2,2-dimethylpropanoate	C_9_H_18_O_2_	5129-37-3	158.24	85	86	84	0.05	0.11	0.07
9	(2,5-dimethyloxan-2-yl)methanol	C_8_H_16_O_2_	54004-46-5	144.21	83	83	82	0.06	0.14	0.16
10	4,6,8-trimethylnon-1-ene	C_12_H_24_	54410-98-9	168.32	89	88	83	0.02	0.01	0.01
11	linoleic acid	C_18_H_32_O_2_	60-33-3	280.45	91	92	93	0.46	0.54	0.89
12	2,4,4-trimethylpentane-1,3-diyl bis(2-methylpropanoate)	C_16_H_30_O_4_	74381-40-1	286.41	95	93	94	1.13	0.61	0.66
13	(S)-falcarinol	C_17_H_24_O	81203-57-8	244.37	91	93	92	1.31	2.35	0.8
14	2-isopropyl-5-methyl-1-heptanol	C_11_H_24_O	91337-07-4	172.31	90	82	87	1.32	0.01	0.04
15	2,4-di-t-butylphenol	C_14_H_22_O	96-76-4	206.32	96	96	96	1.32	0.19	0.42
16	4-methyldecane	C_11_H_24_	2847-72-5	156.31	95	88	-	0.14	0.02	-
17	(E)-7-methyldec-4-ene	C_11_H_22_	62338-48-1	154.29	85	88	-	0.04	0.07	-
18	2,4-diethylheptan-1-ol	C_11_H_24_O	80192-55-8	172.31	88	89	-	1.03	1.86	-
19	2,2'-methylenebis(6-tert-butyl-4-methylphenol)	C_23_H_32_O_2_	119-47-1	340.50	94	-	93	7.88	-	6.69
20	2-isopropyl-5-methyl-1-hexanol	C_10_H_22_O	2051-33-4	158.28	87	-	83	0.01	-	0.01
21	palmitic acid	C_16_H_32_O_2_	57-10-3	256.42	89	-	90	0.96	-	1.15
22	2-methylundecan-1-ol	C_12_H_26_O	10522-26-6	186.33	-	88	85	-	0.05	0.04
23	2,5-dimethyl-2,5-hexanediol	C_8_H_18_O_2_	110-03-2	146.23	-	92	89	-	0.03	0.02
24	2,2,5,5-tetramethyloxolane	C_8_H_16_O	15045-43-9	128.21	-	90	89	-	0.01	0.01
25	1-hexoxyoctane	C_14_H_30_O	17071-54-4	214.39	-	89	88	-	0.02	0.02
26	2,5-dihydroperoxy-2,5-dimethylhexane	C_8_H_18_O_4_	3025-88-5	178.23	-	82	81	-	0.01	0.01
27	palmityl acetate	C_18_H_36_O_2_	629-70-9	284.48	-	91	95	-	0.42	1.13
28	3,7,11-trimethyldodecan-1-ol	C_15_H_32_O	6750-34-1	228.41	-	87	86	-	0.04	0.03
29	4-methylundec-1-ene	C_12_H_24_	74630-39-0	168.32	-	87	86	-	0.01	0.01
30	4-methyl-1-heptanol	C_8_H_18_O	817-91-4	130.23	-	86	87	-	0.02	0.04
31	methyl linoleate	C_19_H_34_O_2_	112-63-0	294.47	91	-	-	0.2	-	-
32	2,6-di-tert-butyl-4-methylphenol	C_15_H_24_O	128-37-0	200.35	83	-	-	0.16	-	-
33	diisononyl phthalate	C_26_H_42_O_4_	20548-62-3	418.61	81	-	-	0.13	-	-
34	didecyl ether	C_20_H_42_O	2456-28-2	298.55	83	-	-	0.01	-	-
35	2-(2-(2-methoxyethoxy)ethoxy)-2-methylpropane	C_9_H_20_O_3_	52788-79-1	176.25	80	-	-	0.05	-	-
36	1-oxacyclotetradeca-4,11-diyne	C_13_H_18_O	6568-32-7	190.28	81	-	-	0.93	-	-
37	2-(1-ethoxyethoxy)-3-methylbutane-1,4-diol	C_9_H_20_O_4_	88481-54-3	192.25	80	-	-	0.01	-	-
38	1-hydroxycyclohexyl phenyl petone	C_13_H_16_O_2_	947-19-3	204.27	95	-	-	1.75	-	-
39	1-dodecanol	C_12_H_26_O	112-53-8	186.33	-	93	-	-	0.09	-
40	(2S,3R)-2-methyl-3-pentyloxirane	C_8_H_16_O	23024-54-6	128.21	-	85	-	-	0.02	-
41	di-tert-butoxy-methane	C_9_H_20_O_2_	2568-93-6	160.25	-	92	-	-	0.01	-
42	2,5-dimethyl-2-hexanol	C_8_H_18_O	3730-60-7	130.23	-	89	-	-	0.01	-
43	dihydrocarveol	C_10_H_18_O	38049-26-2	154.25	-	80	-	-	0.01	-
44	4-methylhept-6-en-3-ol	C_8_H_16_O	53907-71-4	128.21	-	82	-	-	0.01	-
45	Z-9-tetradecenal	C_14_H_26_O	53939-27-8	210.36	-	89	-	-	0.36	-
46	3-methoxy-3-methyl-1-butanol	C_6_H_14_O_2_	56539-66-3	118.17	-	82	-	-	0.02	-
47	2-methyl-2-heptanol	C_8_H_18_O	625-25-2	130.23	-	89	-	-	0.01	-
48	isopropyl laurate	C_15_H_30_O_2_	10233-13-3	242.40	-	-	88	-	-	0.14
49	3,7,11,15-tetramethyl-2-hexadecen-1-ol	C_20_H_40_O	102608-53-7	296.53	-	-	89	-	-	0.12
50	phytol	C_20_H_40_O	150-86-7	296.53	-	-	92	-	-	0.34
51	trans-β-farnesene	C_15_H_24_	18794-84-8	204.35	-	-	81	-	-	0.01
52	pentadec-2-yn-1-ol	C_15_H_28_O	2834-00-6	224.38	-	-	80	-	-	0.01
53	isobutyric acid 2-ethyl-hexyl ester	C_12_H_24_O_2_	35061-61-1	200.32	-	-	82	-	-	0.02
54	2,5-dimethylundec-2-ene	C_13_H_26_	49622-16-4	182.35	-	-	88	-	-	0.07
55	6,10,14-trimethyl-2-pentadecanone	C_18_H_36_O	502-69-2	268.48	-	-	85	-	-	0.03
56	Z-7-tetradecenal	C_14_H_26_O	65128-96-3	210.36	-	-	85	-	-	0.5
57	methyl 2-methoxyprop-2-enoate	C_5_H_8_O_3_	7001-18-5	116.12	-	-	83	-	-	0.05

*-, not detected*.

## Conclusion

Quantitative comparison combined with a non-targeted metabolomics approach was performed to evaluate the chemical differences and similarities between different botanical parts of *P. notoginseng* from different geographical regions. The results demonstrated high similarity in chemical characteristics of the roots and stems with higher content of PPT-type saponins, while they were significantly different from those of the leaves. Geographical location had little effect on the quality of roots but had significant effect on stems and leaves. Specifically, the quality of stems from PuEr City was unstable. In addition, based on a non-targeted metabolomics approach using UHPLC-Q-TOF-MS/MS and GC-MS methods, 52 compounds (including 26 non-volatile and 26 volatile constituents) with VIP > 1 were identified as potential chemical markers to differentiate the different botanical parts of the plant. Taken together, this study elucidates the metabolic analyses of the different botanical parts of *P. notoginseng* obtained from multiple geographical regions, thereby providing a chemical evidence for the rational application of individual parts of this plant.

## Data Availability Statement

The original contributions presented in the study are included in the article/Supplementary Material, further inquiries can be directed to the corresponding author.

## Author Contributions

RS and JH designed the experiment. YC and HO analyzed the experimental data. MG and XC performed the experiment. SW and XH wrote the manuscript. All authors contributed to the article and approved the submitted version.

## Funding

This study was supported by National Natural Science Foundation of China (81673824); National Key R&D Program of China (2021YFD1000202); Yunnan provincial key programs (202102AE090042, 2019ZG00901, 202002AA10007).

## Conflict of Interest

The authors declare that the research was conducted in the absence of any commercial or financial relationships that could be construed as a potential conflict of interest.

## Publisher's Note

All claims expressed in this article are solely those of the authors and do not necessarily represent those of their affiliated organizations, or those of the publisher, the editors and the reviewers. Any product that may be evaluated in this article, or claim that may be made by its manufacturer, is not guaranteed or endorsed by the publisher.
