# Supplementary material for: Quantitative Comparison and Chemical Profile of Different Botanical Parts of Panax notoginseng From Different Regions
Source: Front Nutr. 2022 Apr 27;9:841541. doi: 10.3389/fnut.2022.841541 (PMC9097766; doi:10.3389/fnut.2022.841541)
Supplement: Supplementary file 1 [file Table_1.DOCX]

**Supplementary Table S1.** Sample information of whole plant of *Panax notoginseng* (r: root; s: stem; l: leaf).

| batch | code | origin | batch no. | batch | code | origin | batch no. |
| --- | --- | --- | --- | --- | --- | --- | --- |
| 1 | r1 | PuEr city | 20200423 | 14 | r14 | KunMing city | 20200429 |
|  | s1 |  |  |  | s14 |  |  |
|  | l1 |  |  |  | l14 |  |  |
| 2 | r2 | PuEr city | 20200424 | 15 | r15 | KunMing city | 20200430 |
|  | s2 |  |  |  | s15 |  |  |
|  | l2 |  |  |  | l15 |  |  |
| 3 | r3 | PuEr city | 20200425 | 16 | r16 | KunMing city | 20200504 |
|  | s3 |  |  |  | s16 |  |  |
|  | l3 |  |  |  | l16 |  |  |
| 4 | r4 | PuEr city | 20200503 | 17 | r17 | KunMing city | 20200505 |
|  | s4 |  |  |  | s17 |  |  |
|  | l4 |  |  |  | l17 |  |  |
| 5 | r5 | PuEr city | 20200508 | 18 | r18 | KunMing city | 20200506 |
|  | s5 |  |  |  | s18 |  |  |
|  | l5 |  |  |  | l18 |  |  |
| 6 | r6 | PuEr city | 20200509 | 19 | r19 | KunMing city | 20200511 |
|  | s6 |  |  |  | s19 |  |  |
|  | l6 |  |  |  | l19 |  |  |
| 7 | r7 | PuEr city | 20200510 | 20 | r20 | KunMing city | 20200512 |
|  | s7 |  |  |  | s20 |  |  |
|  | l7 |  |  |  | l20 |  |  |
| 8 | r8 | PuEr city | 20200527 | 21 | r21 | KunMing city | 20200513 |
|  | s8 |  |  |  | s21 |  |  |
|  | l8 |  |  |  | l21 |  |  |
| 9 | r9 | PuEr city | 20200603 | 22 | r22 | KunMing city | 20200520 |
|  | s9 |  |  |  | s22 |  |  |
|  | l9 |  |  |  | l22 |  |  |
| 10 | r10 | WenShan Autonomous Prefecture | 20200426 | 23 | r23 | KunMing city | 20200523 |
|  | s10 |  |  |  | s23 |  |  |
|  | l10 |  |  |  | l23 |  |  |
| 11 | r11 | WenShan Autonomous Prefecture | 20200515 | 24 | r24 | KunMing city | 20200601 |
|  | s11 |  |  |  | s24 |  |  |
|  | l11 |  |  |  | l24 |  |  |
| 12 | r12 | WenShan Autonomous Prefecture | 20200605 | 25 | r25 | KunMing city | 20200602 |
|  | s12 |  |  |  | s25 |  |  |
|  | l12 |  |  |  | l25 |  |  |
| 13 | r13 | KunMing city | 20200428 |  |  |  |  |
|  | s13 |  |  |  |  |  |  |
|  | l13 |  |  |  |  |  |  |

**Supplementary Table S2.** Mass spectrometry parameters of eighteen target saponins.

| saponin | precursor ion (m/z) | product ion (m/z) | fagmentor  (v) | collision  energy (v) | ion mode |
| --- | --- | --- | --- | --- | --- |
| ginsenoside Rf | 799.5 | 637.4 | 280 | 33 | negative |
| ginsenoside Rg1 | 845.5 | 637.3 | 180 | 30 | negative |
| notoginsenoside Fe | 961.5 | 915.6 | 180 | 23 | negative |
| notoginsenoside R1 | 977.5 | 931.6 | 150 | 20 | negative |
| ginsenoside Rg2 | 783.5 | 637.5 | 280 | 31 | negative |
| notoginsenoside Fd | 961.6 | 915.6 | 170 | 23 | negative |
| ginsenoside Re | 945.6 | 637.6 | 300 | 41 | negative |
| ginsenoside Rd | 991.6 | 945.7 | 155 | 20 | negative |
| ginsenoside Rb3 | 1123.4 | 1077.5 | 170 | 20 | negative |
| ginsenoside Rb2 | 1123.4 | 1077.4 | 170 | 20 | negative |
| ginsenoside Rc | 1123.4 | 1077.4 | 175 | 20 | negative |
| ginsenoside Fa | 1239.4 | 1239.4 | 270 | 5 | negative |
| ginsenoside Rb1 | 1107.6 | 1107.6 | 240 | 5 | negative |
| notoginsenoside Fc | 1209.7 | 1077.6 | 245 | 50 | negative |
| ginsenoside Rk1 | 811.5 | 765.6 | 130 | 15 | negative |
| ginsenoside Rg5 | 811.5 | 765.5 | 130 | 15 | negative |
| ginsenoside Rg3 | 829.4 | 783.4 | 145 | 20 | negative |
| ginsenoside F2 | 829.5 | 621.3 | 170 | 28 | negative |

**Supplementary Table S3.** Standard curve regression equation, LLOD and LLOQ of 18 saponins.

| saponin | linear equation | linearity range（ng/mL） | | *r^2^* | LLOQ（ng/mL） | | LLOD（ng/mL） |
| --- | --- | --- | --- | --- | --- | --- | --- |
| notoginsenoside R1 | y = 4306.9464x-12588.2469 | 14-7000 | 0.9992 | | | 3.8 | 1.1 |
| ginsenoside Re | y = 725.2318x-733.9542 | 2-1000 | 0.9996 | | | 0.1 | 0.03 |
| ginsenoside Rg1 | y = 1041.4443x-2495.7098 | 24-12000 | 0.9994 | | | 0.03 | 0.01 |
| ginsenoside Fa | y = 3694.1201x-17110.5285 | 30-15000 | 0.9991 | | | 2.2 | 0.7 |
| ginsenoside Rf | y = 700.8444x-34.7449 | 0.2-100 | 0.9995 | | | 0.04 | 0.01 |
| ginsenoside Rb1 | y = 5530.1440x-55127.8407 | 30-15000 | 0.9994 | | | 1.4 | 0.4 |
| ginsenoside Rc | y = 4671.7822x+86288.3561 | 36-18000 | 0.9995 | | | 0.9 | 0.3 |
| ginsenoside Rg2 | y = 557.9440x-353.3671 | 1.6-800 | 0.9995 | | | 0.8 | 0.2 |
| notoginsenoside Fc | y = 296.5986x-3165.1077 | 36-18000 | 0.9992 | | | 0.2 | 0.05 |
| ginsenoside Rb2 | y = 6324.2290x+3765.4786 | 30-15000 | 0.9993 | | | 1.0 | 0.3 |
| ginsenoside Rb3 | y = 4676.5084x+147028.2815 | 60-30000 | 0.9994 | | | 1.2 | 0.4 |
| ginsenoside Rd | y = 3903.1331x-6092.0015 | 10-5000 | 0.9994 | | | 0.9 | 0.3 |
| notoginsenoside Fe | y = 5072.1664x+42348.0830 | 36-18000 | 0.9991 | | | 2.8 | 0.8 |
| notoginsenoside Fd | y = 5550.6778x+151898.0386 | 64-32000 | 0.9993 | | | 3.5 | 1.1 |
| ginsenoside F2 | y = 1234.3569x+455.3127 | 8-4000 | 0.9993 | | | 0.02 | 0.01 |
| ginsenoside Rg3 | y = 17636.5674x-7984.6410 | 1-500 | 0.9994 | | | 0.8 | 0.2 |
| ginsenoside Rk1 | y = 6854.7347x+386.2999 | 0.25-125 | 0.9994 | | | 0.2 | 0.07 |
| ginsenoside Rg5 | y = 23940.6764x-3191.2651 | 0.25-125 | 0.9996 | | | 0.2 | 0.07 |

**Supplementary Table S4.** The contents of 18 saponins in different parts of *P. notoginseng* from different areas (μg/g) (n = 3).

| batch | part | Saponin | | | | | | | | | | | | | | | | | | |
| --- | --- | --- | --- | --- | --- | --- | --- | --- | --- | --- | --- | --- | --- | --- | --- | --- | --- | --- | --- | --- |
|  |  | N-R1 | G-Re | G-Rg1 | G-Fa | G-Rf | G-Rb1 | G-Rc | G-Rg2 | N-Fc | G-Rb2 | G-Rb3 | G-Rd | N-Fe | N-Fd | G-F2 | G-Rg3 | G-Rk1 | G-Rg5 |  |
| 1 | root | 1251.61 | 197.15 | 6481.15 | 211.99 | 13.22 | 3601.83 | -- | 228.25 | 37.35 | 34.35 | 69.02 | 1068.54 | -- | 67.92 | 416.67 | 10.37 | 0.32 | 0.27 |  |
|  | stem | 288.15 | 55.66 | 1972.91 | 669.14 | 3.00 | 1541.41 | 256.21 | 38.61 | 725.83 | 126.06 | 573.61 | 310.23 | 1267.69 | 3178.57 | 862.91 | 9.93 | 0.26 | 0.26 |  |
|  | leaf | 15.44 | 17.31 | 94.72 | 2863.22 | 0.28 | 196.31 | 1485.94 | 6.15 | 5471.14 | 528.73 | 2984.47 | 49.55 | 11379.79 | 18957.58 | 1091.77 | 23.47 | 0.57 | 0.38 |  |
| 2 | root | 921.66 | 247.59 | 6219.39 | 132.69 | 5.90 | 4396.87 | -- | 162.75 | 37.02 | 35.85 | -- | 556.71 | -- | -- | 149.69 | 8.94 | 0.49 | 0.37 |  |
|  | stem | 210.70 | 110.11 | 2909.96 | 484.22 | 3.13 | 2131.93 | 974.19 | 51.18 | 428.60 | 360.44 | 1960.94 | 342.44 | 353.86 | 828.13 | 119.89 | 7.61 | 0.28 | 0.25 |  |
|  | leaf | 15.44 | 17.31 | 94.72 | 2863.22 | 0.28 | 196.31 | 1485.94 | 6.15 | 5471.14 | 528.73 | 2984.47 | 49.55 | 11379.79 | 18957.58 | 1091.77 | 23.47 | 0.57 | 0.38 |  |
| 3 | root | 412.85 | 263.48 | 3530.91 | 109.39 | 5.21 | 2879.94 | -- | 195.78 | 36.99 | 31.12 | 63.80 | 686.79 | -- | 67.45 | 93.12 | 7.67 | 0.32 | 0.25 |  |
|  | stem | 208.36 | 128.11 | 2079.64 | 559.13 | 1.51 | 1594.01 | 131.81 | 40.96 | 628.76 | 83.20 | 272.97 | 186.41 | 1407.77 | 3327.51 | 483.19 | 8.30 | 0.25 | 0.25 |  |
|  | leaf | -- | 14.90 | 24.44 | 906.69 | 0.50 | 34.22 | 491.78 | 6.65 | 5571.06 | 169.85 | 1098.64 | 41.41 | 9195.89 | 16806.42 | 747.88 | 12.05 | 0.35 | 0.34 |  |
| 4 | root | 636.71 | 103.43 | 2975.55 | 95.50 | 6.43 | 2987.69 | -- | 89.17 | 36.46 | 31.08 | -- | 540.43 | 37.62 | 66.11 | 206.59 | 8.50 | 0.39 | 0.29 |  |
|  | stem | 912.79 | 120.52 | 2536.07 | 523.92 | 5.60 | 3419.50 | 87.61 | 64.33 | 162.50 | 62.23 | 207.58 | 442.11 | 471.25 | 1217.39 | 432.53 | 14.77 | 0.59 | 0.40 |  |
|  | leaf | 14.68 | 6.57 | 73.19 | 1802.68 | 0.26 | 60.80 | 744.79 | 3.86 | 4409.87 | 224.69 | 1810.65 | 52.34 | 10395.21 | 18420.28 | 1295.32 | 22.81 | 0.45 | 0.47 |  |
| 5 | root | 1113.38 | 235.10 | 4435.71 | 108.09 | 6.09 | 2360.97 | -- | 155.66 | 36.51 | 36.67 | -- | 554.44 | -- | 66.04 | 235.08 | 8.54 | 0.42 | 0.27 |  |
|  | stem | 1510.78 | 210.02 | 3603.57 | 550.64 | 3.30 | 1604.11 | 105.00 | 42.03 | 677.54 | 79.89 | 251.33 | 242.43 | 1623.08 | 3889.19 | 763.64 | 8.95 | 0.42 | 0.31 |  |
|  | leaf | -- | 10.06 | 50.42 | 822.74 | 0.25 | 57.86 | 815.65 | 6.11 | 8803.23 | 337.58 | 2131.87 | 138.89 | 13312.18 | 23125.30 | 2836.81 | 13.84 | 2.30 | 1.27 |  |
| 6 | root | 1404.43 | 340.33 | 3100.74 | 159.44 | 10.94 | 3615.06 | -- | 242.15 | 36.81 | 31.96 | -- | 647.36 | -- | -- | 116.79 | 6.17 | 0.51 | 0.31 |  |
|  | stem | 1180.36 | 424.39 | 4855.69 | 611.65 | 5.64 | 1996.03 | 2.95 | 167.82 | 399.38 | 31.96 | 61.97 | 203.65 | 1203.16 | 2999.79 | 520.58 | 7.92 | 0.32 | 0.26 |  |
|  | leaf | 53.23 | 11.66 | 60.17 | 2187.09 | 0.26 | 47.00 | 338.36 | 7.30 | 5629.32 | 151.19 | 733.48 | 21.49 | 11348.99 | 19570.33 | 1047.52 | 16.09 | 0.29 | 0.30 |  |
| 7 | root | 794.47 | 356.35 | 5503.48 | 172.86 | 7.59 | 5347.86 | -- | 311.93 | 36.62 | 42.12 | 61.23 | 1403.39 | -- | -- | 100.07 | 147.54 | 0.86 | 0.37 |  |
|  | stem | 1449.50 | 649.61 | 7390.71 | 660.06 | 9.49 | 9028.31 | 262.85 | 376.53 | 323.77 | 300.23 | 544.13 | 1050.53 | 749.94 | 1782.22 | 352.70 | 21.75 | 0.72 | 0.44 |  |
|  | leaf | -- | 5.94 | 24.70 | 4599.79 | 0.39 | 58.35 | 454.79 | 4.21 | 6177.69 | 189.13 | 1178.74 | 28.76 | 13126.18 | 22637.03 | 976.92 | 38.23 | 0.44 | 0.28 |  |
| 8 | root | 1120.95 | 321.01 | 5871.40 | 212.81 | 11.23 | 5205.23 | -- | 391.81 | -- | 31.33 | 65.15 | 615.85 | -- | 66.50 | 120.51 | 10.08 | 1.43 | 0.70 |  |
|  | stem | 356.52 | 102.32 | 2470.52 | 821.48 | 1.86 | 1732.47 | 63.46 | 58.32 | 434.47 | 46.12 | 140.44 | 134.35 | 996.28 | 2704.75 | 445.53 | 12.61 | 0.40 | 0.26 |  |
|  | leaf | -- | 3.35 | 24.24 | 2012.07 | 0.36 | 48.79 | 811.91 | 4.23 | 6108.85 | 261.31 | 1764.39 | 90.23 | 11494.38 | 20652.79 | 1281.00 | 30.07 | 0.71 | 0.46 |  |
| 9 | root | 714.47 | 134.30 | 4105.16 | 140.19 | 8.76 | 6198.93 | -- | 156.39 | -- | 35.65 | -- | 1257.60 | -- | -- | 89.35 | 10.78 | 1.65 | 0.79 |  |
|  | stem | 525.91 | 150.40 | 3327.58 | 506.34 | 5.51 | 5173.10 | -- | 94.39 | 216.49 | 83.65 | 61.10 | 735.80 | 903.89 | 2239.75 | 352.23 | 14.37 | 0.64 | 0.46 |  |
|  | leaf | 14.24 | 31.52 | 43.42 | 3470.38 | 0.67 | 43.44 | 251.22 | 13.10 | 6209.56 | 88.97 | 627.91 | 20.08 | 12529.89 | 22569.89 | 942.36 | 29.18 | 0.28 | 0.25 |  |
| 10 | root | 1221.78 | 393.07 | 6929.15 | 208.08 | 10.90 | 6271.01 | -- | 338.12 | 36.25 | 31.51 | 64.98 | 1284.19 | -- | -- | 235.62 | 9.68 | 0.44 | 0.26 |  |
|  | stem | 26.74 | 19.24 | 126.92 | 924.61 | 0.29 | 3607.72 | 1234.54 | 5.69 | 617.72 | 1051.68 | 3469.25 | 247.84 | 92.64 | 296.51 | 50.85 | 3.12 | 0.26 | 0.25 |  |
|  | leaf | -- | 11.99 | 24.94 | 2775.02 | 0.26 | 2193.78 | 7720.46 | 4.41 | 5610.34 | 4291.41 | 15932.08 | 133.67 | 2202.21 | 6416.60 | 59.64 | 7.99 | 0.79 | 0.33 |  |
| 11 | root | 2293.25 | 452.05 | 6375.58 | 266.38 | 18.24 | 7975.74 | -- | 484.31 | -- | 32.70 | -- | 1792.87 | -- | -- | 168.97 | 12.51 | 0.30 | 0.25 |  |
|  | stem | 86.74 | 31.88 | 386.10 | 1112.37 | 0.79 | 3297.44 | 211.09 | 22.05 | 162.18 | 183.23 | 458.75 | 190.24 | -- | 66.64 | 14.13 | 2.14 | 0.26 | 0.25 |  |
|  | leaf | -- | 5.43 | 24.68 | 4063.09 | 0.21 | 2626.91 | 10648.44 | 2.91 | 6626.86 | 5669.33 | 17321.95 | 229.74 | 951.84 | 2171.21 | 10.28 | 8.17 | 0.93 | 0.37 |  |
| 12 | root | 997.12 | 475.09 | 7575.00 | 260.33 | 11.81 | 8558.32 | 59.84 | 535.52 | 82.99 | 59.81 | 208.36 | 1439.46 | 86.50 | 248.42 | 69.03 | 13.22 | 0.49 | 0.27 |  |
|  | stem | 44.34 | 27.38 | 209.26 | 666.40 | 0.44 | 3409.41 | 236.13 | 9.34 | 99.09 | 252.55 | 615.76 | 153.07 | -- | -- | 12.78 | 1.69 | 0.27 | 0.26 |  |
|  | leaf | 16.54 | 33.01 | 41.84 | 5340.67 | 0.28 | 3840.45 | 12806.01 | 4.49 | 8635.48 | 8021.15 | 20623.37 | 364.61 | 1269.94 | 3048.33 | 17.54 | 14.88 | 1.48 | 0.58 |  |
| 13 | root | 5185.11 | 283.18 | 7855.90 | 370.16 | 21.38 | 9387.64 | -- | 133.15 | 36.51 | 66.04 | -- | 1518.88 | -- | -- | 45.36 | 11.80 | 0.46 | 0.26 |  |
|  | stem | 41.36 | 17.89 | 183.97 | 1129.94 | 0.23 | 2264.00 | 264.37 | 4.18 | 297.33 | 280.55 | 691.83 | 108.89 | 37.99 | 68.63 | 8.24 | 1.79 | 0.26 | 0.26 |  |
|  | leaf | 14.75 | 9.51 | 49.27 | 7376.13 | 0.21 | 4086.35 | 12986.73 | 3.05 | 11587.15 | 10444.02 | 22093.30 | 239.68 | 433.63 | 1029.46 | 8.55 | 11.31 | 1.12 | 0.42 |  |
| 14 | root | 878.83 | 159.05 | 3153.31 | 79.39 | 5.79 | 3702.36 | -- | 117.04 | 36.44 | 34.72 | 65.00 | 699.12 | -- | -- | 67.31 | 6.10 | 0.27 | 0.26 |  |
|  | stem | 48.22 | 69.93 | 875.19 | 754.11 | 0.33 | 3642.91 | 394.28 | 5.73 | 165.40 | 319.53 | 1018.18 | 80.55 | -- | -- | 9.82 | 1.62 | 0.26 | 0.25 |  |
|  | leaf | 100.88 | 44.57 | 342.60 | 5032.54 | 0.29 | 6016.70 | 12850.87 | 7.26 | 6599.90 | 8949.98 | 21117.29 | 207.57 | 1633.51 | 4459.03 | 45.17 | 17.48 | 1.32 | 0.51 |  |
| 15 | root | 2020.60 | 321.54 | 7307.50 | 173.84 | 17.70 | 7657.53 | -- | 414.28 | 36.35 | 32.02 | -- | 1352.52 | -- | -- | 76.52 | 8.16 | 0.26 | 0.25 |  |
|  | stem | 216.98 | 30.84 | 1458.75 | 221.43 | 2.24 | 1995.94 | -- | 13.70 | 36.58 | 64.15 | 61.37 | 218.31 | -- | -- | 8.20 | 1.35 | 0.27 | 0.25 |  |
|  | leaf | 14.31 | 9.83 | 38.52 | 3591.00 | 0.22 | 3167.33 | 13016.45 | 4.27 | 6303.75 | 7899.26 | 19557.51 | 209.29 | 378.01 | 703.81 | 8.23 | 8.57 | 1.08 | 0.40 |  |
| 16 | root | 2408.24 | 316.68 | 7555.82 | 268.14 | 14.12 | 6803.37 | -- | 256.00 | 36.48 | 35.37 | -- | 1297.61 | -- | -- | 105.91 | 6.70 | 0.27 | 0.25 |  |
|  | stem | 33.60 | 27.81 | 248.48 | 1349.41 | 0.24 | 4795.75 | 841.28 | 4.72 | 274.61 | 538.09 | 2023.50 | 255.47 | -- | -- | 8.02 | 3.66 | 0.26 | 0.25 |  |
|  | leaf | 19.71 | 20.01 | 120.71 | 7183.33 | 0.25 | 6634.73 | 13895.99 | 3.92 | 8876.22 | 10069.28 | 23682.57 | 545.91 | 656.24 | 1727.00 | 8.82 | 24.31 | 1.59 | 0.59 |  |
| 17 | root | 1674.88 | 292.13 | 6733.70 | 192.34 | 13.70 | 5873.11 | -- | 209.10 | 36.57 | 34.30 | 63.25 | 902.17 | -- | -- | 129.46 | 7.02 | 0.33 | 0.25 |  |
|  | stem | 23.85 | 20.31 | 89.62 | 794.28 | 0.27 | 3316.09 | 172.38 | 3.51 | 89.20 | 175.94 | 489.27 | 64.35 | -- | -- | 9.41 | 1.52 | 0.26 | 0.26 |  |
|  | leaf | 16.75 | 16.07 | 73.44 | 6301.20 | 0.46 | 5230.65 | 13432.59 | 4.83 | 7148.70 | 8679.40 | 23290.62 | 148.82 | 2942.08 | 8423.63 | 16.06 | 12.29 | 0.85 | 0.42 |  |
| 18 | root | 411.46 | 74.06 | 2081.32 | 43.36 | 2.77 | 2334.59 | -- | 47.40 | 36.47 | 30.65 | 62.89 | 257.36 | -- | -- | 20.28 | 2.38 | 0.25 | 0.25 |  |
|  | stem | 160.16 | 48.95 | 685.66 | 581.75 | 0.98 | 4382.71 | 343.40 | 14.37 | 100.05 | 300.41 | 878.23 | 126.95 | -- | -- | 8.08 | 2.01 | 0.35 | 0.26 |  |
|  | leaf | 53.91 | 36.33 | 274.31 | 5620.23 | 0.24 | 7516.32 | 16110.22 | 9.77 | 5677.42 | 11053.44 | 25893.79 | 326.82 | 274.76 | 665.50 | 23.78 | 16.61 | 1.71 | 0.67 |  |
| 19 | root | 747.94 | 140.19 | 2500.68 | 62.70 | 5.41 | 2675.74 | 37.17 | 87.50 | 36.68 | 30.85 | 68.78 | 398.56 | -- | -- | 49.89 | 3.04 | 0.30 | 0.25 |  |
|  | stem | 97.06 | 54.80 | 391.88 | 871.31 | 0.64 | 5971.63 | 335.88 | 11.99 | 125.04 | 439.53 | 838.86 | 192.47 | -- | -- | 8.86 | 2.99 | 0.50 | 0.28 |  |
|  | leaf | 69.11 | 45.69 | 154.99 | 6932.65 | 0.31 | 9245.96 | 17354.65 | 10.50 | 5810.33 | 13421.98 | 28402.07 | 773.25 | 249.12 | 627.79 | 12.30 | 24.05 | 2.49 | 0.84 |  |
| 20 | root | 289.73 | 99.04 | 1404.06 | 43.28 | 3.59 | 414.90 | 58.38 | 94.62 | 36.54 | 34.38 | 228.88 | 71.36 | 46.86 | 272.03 | 12.11 | 2.07 | 0.25 | 0.25 |  |
|  | stem | 348.21 | 72.44 | 1830.66 | 649.12 | 1.56 | 2502.92 | 183.70 | 24.52 | 157.55 | 324.60 | 478.68 | 329.30 | 36.63 | 70.96 | 26.66 | 2.50 | 0.30 | 0.25 |  |
|  | leaf | 14.11 | 3.02 | 24.44 | 4542.22 | 0.21 | 2369.85 | 12517.60 | 2.66 | 12645.64 | 8569.89 | 21363.73 | 429.04 | 839.69 | 1958.12 | 43.14 | 10.68 | 1.11 | 0.40 |  |
| 21 | root | 955.18 | 290.50 | 6529.70 | 99.19 | 12.62 | 3507.95 | 41.22 | 405.56 | 36.49 | 33.11 | 154.94 | 492.41 | -- | 72.81 | 39.80 | 6.33 | 0.31 | 0.26 |  |
|  | stem | 84.59 | 28.77 | 348.50 | 1047.54 | 0.55 | 3239.73 | 368.85 | 8.40 | 225.18 | 380.94 | 1026.10 | 254.69 | -- | -- | 23.25 | 2.09 | 0.26 | 0.25 |  |
|  | leaf | 14.37 | 8.81 | 26.73 | 4458.66 | 0.20 | 2937.24 | 11560.00 | 4.51 | 9522.97 | 7102.75 | 20414.76 | 394.67 | 572.37 | 1446.08 | 35.95 | 9.56 | 0.75 | 0.32 |  |
| 22 | root | 1348.62 | 333.73 | 5988.89 | 360.56 | 14.89 | 4257.16 | -- | 268.41 | -- | 31.24 | 61.37 | 597.33 | -- | -- | 12.27 | 7.90 | 0.26 | 0.26 |  |
|  | stem | 20.68 | 22.02 | 50.81 | 492.43 | 0.28 | 2099.13 | 128.59 | 2.56 | 90.77 | 123.04 | 397.03 | 119.80 | -- | -- | 19.71 | 1.19 | 0.26 | 0.25 |  |
|  | leaf | 14.32 | 13.78 | 27.76 | 3795.95 | 0.24 | 2444.76 | 13602.84 | 4.77 | 9506.15 | 7268.64 | 21120.59 | 303.79 | 981.19 | 2155.15 | 28.36 | 10.77 | 1.17 | 0.39 |  |
| 23 | root | 116.50 | 156.28 | 1410.45 | 31.05 | 1.65 | 337.42 | 86.07 | 167.17 | 36.13 | 39.48 | 298.44 | 62.05 | 76.01 | 373.45 | 40.51 | 4.70 | 0.26 | 0.25 |  |
|  | stem | 436.58 | 93.54 | 3504.87 | 692.47 | 4.18 | 5334.98 | 1232.05 | 37.61 | 394.07 | 798.40 | 2788.48 | 726.65 | 50.33 | 145.70 | 79.14 | 4.95 | 0.38 | 0.26 |  |
|  | leaf | 14.78 | 8.08 | 46.15 | 4997.34 | 0.22 | 7078.78 | 17170.79 | 5.40 | 7851.09 | 12191.63 | 27646.89 | 706.22 | 242.42 | 531.33 | 19.15 | 11.35 | 1.29 | 0.50 |  |
| 24 | root | 590.69 | 248.23 | 2577.24 | 160.28 | 5.81 | 1030.78 | 186.27 | 194.33 | 36.16 | 54.83 | 531.79 | 145.84 | 319.79 | 1155.96 | 69.62 | 12.11 | 0.43 | 0.29 |  |
|  | stem | 262.45 | 35.83 | 613.41 | 641.53 | 0.79 | 3767.08 | 410.75 | 7.03 | 180.67 | 298.54 | 986.71 | 180.71 | -- | -- | 8.63 | 2.47 | 0.28 | 0.26 |  |
|  | leaf | 14.42 | 8.52 | 26.46 | 7407.22 | 0.22 | 7868.42 | 17611.20 | 2.83 | 9900.07 | 13202.75 | 28861.23 | 420.97 | 1674.84 | 4086.36 | 12.86 | 25.81 | 1.82 | 0.65 |  |
| 25 | root | 1206.94 | 301.79 | 5308.24 | 185.97 | 8.16 | 4751.52 | 72.64 | 217.00 | 48.93 | 61.29 | 189.11 | 770.44 | -- | -- | 299.03 | 7.87 | 0.25 | 0.26 |  |
|  | stem | 99.88 | 37.22 | 599.57 | 525.13 | 0.93 | 2686.15 | 737.97 | 16.62 | 227.56 | 392.35 | 1627.79 | 152.89 | 42.83 | 77.52 | 16.99 | 2.97 | 0.33 | 0.26 |  |
|  | leaf | -- | 11.64 | 28.45 | 4839.13 | 0.76 | 3703.85 | 15631.02 | 4.11 | 7794.80 | 8504.32 | 24887.91 | 235.55 | 1179.11 | 2576.49 | 12.44 | 14.76 | 1.26 | 0.45 |  |

--, Not detected.

**Supplementary Table S5**. Differential compounds responsible for differentiation of root, stem and leaf.

| no. | retention time (min) | precursor ion | fragment ion | loading form | possible compound | molecular formula | diff (ppm) |
| --- | --- | --- | --- | --- | --- | --- | --- |
| 1 | 0.92 | 175.1182 | 156.0748, 133.0634 | [M+H]^+^ | l-arginine | C_6_H_14_N_4_O_2_ | 4.29 |
| 2 | 1.54 | 132.1011 | 69.0694 | [M+H]^+^ | l-isoleucine | C_6_H_13_NO_2_ | 6.17 |
| 3 | 10.46 | 977.5289 | 931.5225, 799.4802, 637.4292, 475.3767 | [M+COOH]^-^ | notoginsenoside R1 | C_47_H_80_O_18_ | 3.56 |
| 4 | 10.94 | 845.4883 | 799.4805, 637.4282, 475.3764 | [M+COOH]^-^ | ginsenoside Rg1 | C_42_H_72_O_14_ | 7.23 |
| 5 | 10.96 | 991.5432 | 945.5362, 799.4810, 637.2863, 475.3768 | [M+COOH]^-^ | ginsenoside Re | C_42_H_82_O_18_ | 5.28 |
| 6 | 11.47 | 885.4825 | 841.4940 | [M-H]^-^ | malonyl-ginsenoside Rg1/isomer | C_45_H_74_O_17_ | 3.49 |
| 7 | 12.34 | 1167.5718 | 1157.5445, 1121.5667 | [M+COOH]^-^ | notoginsenoside B | C_54_H_90_O_24_ | 3.09 |
| 8 | 13.48 | 1371.6727 | 1239.6115, 1107.6055, 945.5295, 783.4825, 621.4325, 459.3796 | [M-H]^-^ | notoginsenoside D/T | C_64_H_108_O_31_ | -4.62 |
| 9 | 14.81 | 1269.6467 | 1305.6202, 1107.5857, 945.5341, 783.4812, 621.4331, 459.3794 | [M-H]^-^ | ginsenoside Ra0 | C_60_H_102_O_28_ | 3.28 |
| 10 | 15.23 | 815.4758 | 805.4471, 769.4710 | [M+COOH]^-^ | notoginsenoside R2/isomer | C_41_H_70_O_13_ | 4.60 |
| 11 | 15.31 | 1093.6056 | 637.4277, 475.3758 | [M-H]^-^ | yesanchinoside H | C_53_H_90_O_23_ | 3.69 |
| 12 | 16.31 | 1107.5921 | 783.4887, 621.4337, 459.3793 | [M-H]^-^ | ginsenoside Rb1 | C_54_H_92_O_23_ | 2.42 |
| 13 | 16.36 | 1239.6320 | 1107.5920, 945.5385, 783.4858, 621.4334 | [M-H]^-^ | notoginsenoside R4 | C_59_H_100_O_27_ | 1.58 |
| 14 | 16.44 | 793.4678 | 441.3707, 423.3606 | [M+Na]^+^ | ginsenoside F5 | C_41_H_70_O_13_ | 3.61 |
| 15 | 17.27 | 1077.5810 | 945.5368, 783.4848, 621.4334, 459.3796 | [M+COOH]^-^ | ginsenoside Rc | C_53_H_90_O_22_ | 2.89 |
| 16 | 17.42 | 1209.6226 | 1077.5786, 945.5382, 783.4844, 621.4332, 459.3799 | [M-H]^-^ | notoginsenoside Fc | C_58_H_98_O_26_ | 2.39 |
| 17 | 18.45 | 1077.5810 | 945.5372, 783.4855, 621.4338, 459.3796 | [M-H]^-^ | ginsenoside Rb2 | C_53_H_90_O_22_ | 1.21 |
| 18 | 18.81 | 1077.5814 | 783.4665, 621.4338, 459.3812 | [M-H]^-^ | ginsenoside Rb3 | C_53_H_9_0O_22_ | 2.82 |
| 19 | 21.01 | 991.5453 | 945.5372, 783.4864, 621.4324, 459.3804, 375.2823 | [M+COOH]^-^ | ginsenoside Rd | C_48_H_82_O_18_ | 2.70 |
| 20 | 21.68 | 1031.5377 | 987.5473 | [M-H]^-^ | malonyl-ginsenoside Rd/isomer | C_51_H_84_O_21_ | 3.95 |
| 21 | 22.21 | 987.5476 | 945.5352, 783.4858, 621.4326, 459.3826 | [M-H]^-^ | pseudoginsenoside Rc1 | C_50_H_84_O_19_ | 6.21 |
| 22 | 23.36 | 991.5442 | 981.5115, 945.5362, 783.4858, 621.4307, 459.3774 | [M+COOH]^-^ | gypenoside ⅩⅦ | C_48_H_82_O_18_ | 4.32 |
| 23 | 25.04 | 961.5355 | 915.5261, 621.4340, 459.3814 | [M+COOH]^-^ | notoginsenoside Fe | C_47_H_80_O_17_ | 2.65 |
| 24 | 26.65 | 961.5353 | 915.5269, 783.4862, 621.4337, 459.3848 | [M+COOH]^-^ | notoginsenoside Fd | C_47_H_80_O_17_ | 2.75 |
| 25 | 28.12 | 955.4846 | 793.4340 | [M-H]^-^ | ginsenoside Ro | C_48_H_76_O_19_ | 2.83 |
| 26 | 29.59 | 667.4374 | 621.3441, 459.3856 | [M+COOH]^-^ | ginsenoside Rh2 | C_36_H_62_O_8_ | 7.84 |

**Supplementary Table S6.** Potential volatile markers responsible for differentiation of root, stem and leaf of *P. notoginseng*.

| no. | retention time (min) | compound | molecular formula | CAS | m/z | similarity |
| --- | --- | --- | --- | --- | --- | --- |
| 1 | 4.35 | 2,4-dimethyl-1-heptene | C_9_H_18_ | 19549-87-2 | 126.24 | 97 |
| 2 | 4.83 | 2-hexyl-1-decanol | C_16_H_34_O | 2425-77-6 | 242.44 | 87 |
| 3 | 4.91 | 11-methyldodecan-1-ol | C_13_H_28_O | 27458-92-0 | 200.36 | 89 |
| 4 | 5.32 | 8-methylnonyl methacrylate | C_14_H_26_O_2_ | 29964-84-9 | 226.36 | 89 |
| 5 | 6.79 | 4,6,8-trimethylnon-1-ene | C_12_H_24_ | 54410-98-9 | 168.32 | 89 |
| 6 | 7.61 | butyl 2,2-dimethylpropanoate | C_9_H_18_O_2_ | 5129-37-3 | 158.24 | 86 |
| 7 | 9.15 | linoleic acid | C_18_H_32_O_2_ | 60-33-3 | 226.36 | 93 |
| 8 | 11.65 | 2,4-diethylheptan-1-ol | C_11_H_24_O | 80192-55-8 | 172.31 | 89 |
| 9 | 12.78 | 2-isopropyl-5-methyl-1-hexanol | C_10_H_22_O | 2051-33-4 | 158.28 | 87 |
| 10 | 13.81 | palmitic acid | C_16_H_32_O_2_ | 57-10-3 | 256.42 | 90 |
| 11 | 14.02 | dihydrocarveol | C_10_H_18_O | 38049-26-2 | 154.25 | 80 |
| 12 | 16.90 | palmityl acetate | C_18_H_36_O_2_ | 629-70-9 | 284.48 | 95 |
| 13 | 17.45 | 4-methyl-1-heptanol | C_8_H_18_O | 817-91-4 | 130.23 | 87 |
| 14 | 17.67 | z-9-tetradecenal | C_14_H_26_O | 53939-27-8 | 210.36 | 89 |
| 15 | 17.71 | methyl linoleate | C_19_H_34_O_2_ | 112-63-0 | 294.47 | 91 |
| 16 | 19.10 | diisononyl phthalate | C_26_H_42_O_4_ | 20548-62-3 | 418.61 | 81 |
| 17 | 19.77 | 1-hydroxycyclohexyl phenyl ketone | C_13_H_16_O_2_ | 947-19-3 | 204.27 | 95 |
| 18 | 20.93 | di-tert-butoxy-methane | C_9_H_20_O_2_ | 2568-93-6 | 160.25 | 92 |
| 19 | 21.18 | 1-dodecanol | C_12_H_26_O | 112-53-8 | 186.33 | 93 |
| 20 | 28.06 | (S)-falcarinol | C_17_H_24_O | 81203-57-8 | 244.37 | 93 |
| 21 | 30.74 | 2,5-dimethyl-2-hexanol | C_8_H_18_O | 3730-60-7 | 130.23 | 89 |
| 22 | 30.92 | 2-methyl-2-heptanol | C_8_H_18_O | 625-25-2 | 130.23 | 89 |
| 23 | 31.71 | isopropyl laurate | C_15_H_30_O_2_ | 10233-13-3 | 242.40 | 88 |
| 24 | 33.18 | 3,7,11,15-tetramethyl-2-hexadecen-1-ol | C_20_H_40_O | 102608-53-7 | 296.53 | 89 |
| 25 | 33.22 | trans-β-farnesene | C_15_H_24_ | 18794-84-8 | 204.35 | 81 |
| 26 | 39.37 | 6,10,14-trimethyl-2-pentadecanone | C_18_H_36_O | 502-69-2 | 268.48 | 85 |


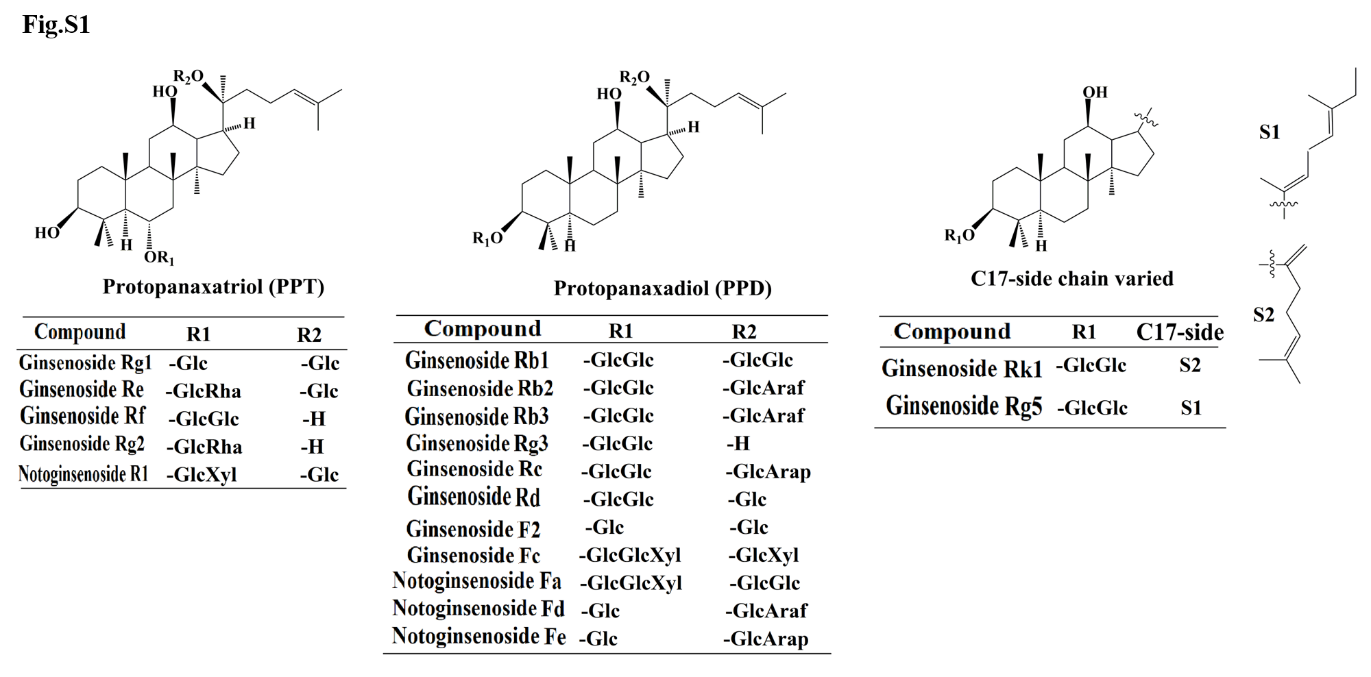


**Supplementary Fig. S1.** The structures of 18 investigated saponins.


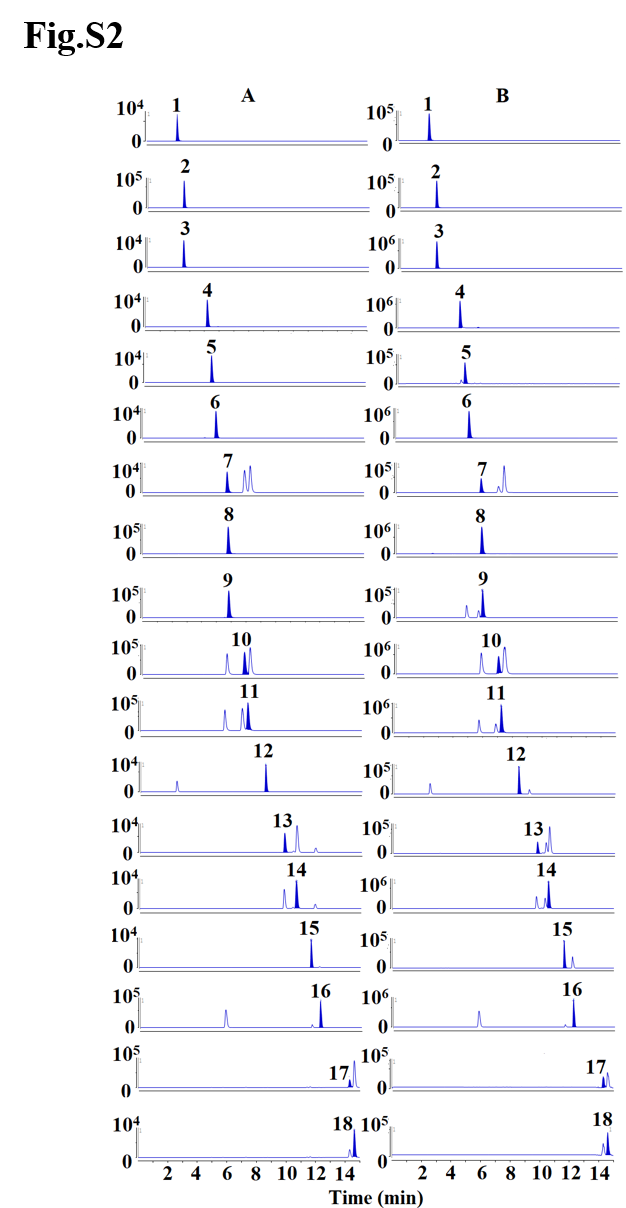


**Supplementary Fig. S2.** MRM of samples (A) and standards (B). Peaks are as follows: 1. N-R1; 2. G-Re; 3. G-Rg1; 4. G-Fa; 5. G-Rf; 6. G-Rb1; 7. G-Rc; 8. G-Rg2; 9. N-Fc; 10. G-Rb2; 11. G-Rb3; 12. G-Rd; 13. N-Fe; 14. N-Fd; 15. G-F2; 16. G-Rg3; 17. G-Rk1; 18. G-Rg5.


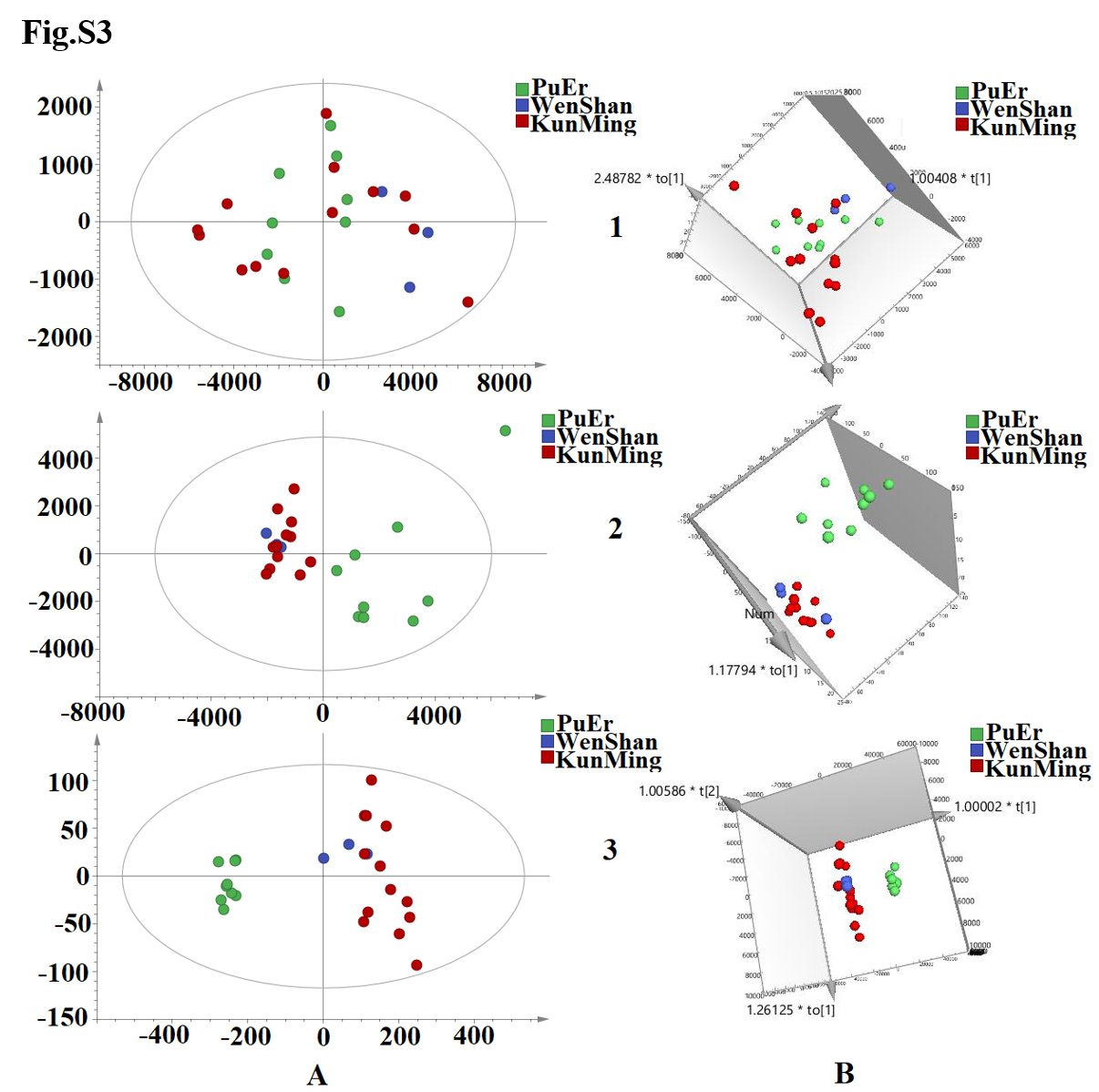


**Supplementary Fig. S3.** PCA scores plot (A) and OPLS-DA scores plot (B) of quantitative data of root (1), stem (2) and leaf (3) from different origins.


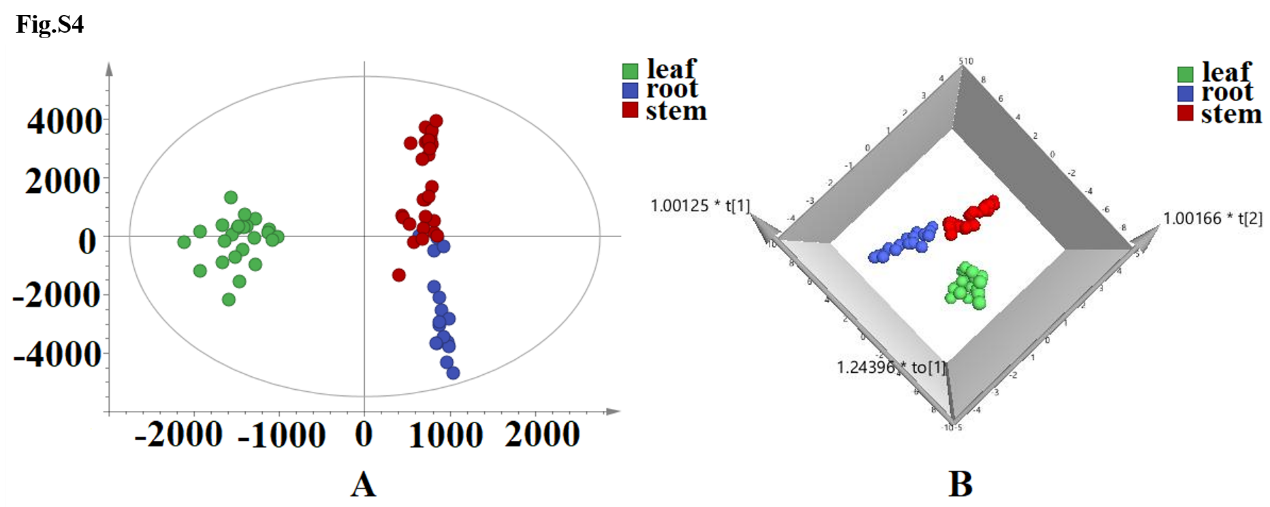


**Supplementary Fig. S4.** PCA scores plot (A) and OPLS-DA scores plot (B) of quantitative data of root, stem and leaf of *P. notoginseng*.


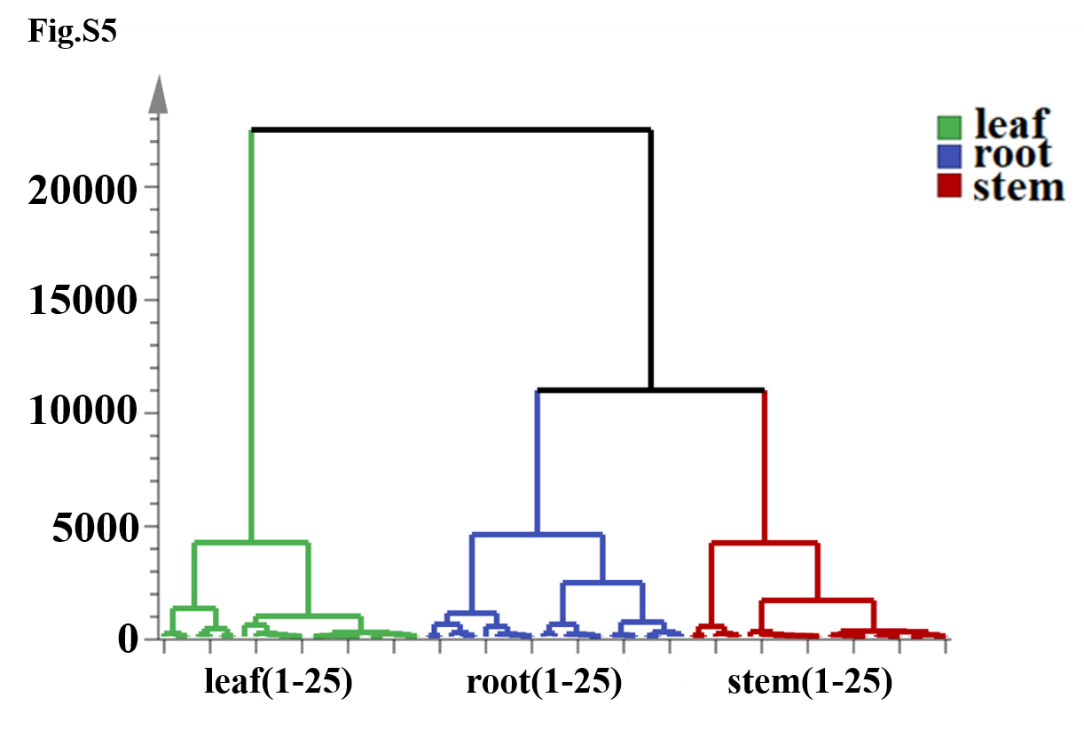


**Supplementary Fig. S5.** HCA dendrogram of UHPLC-Q-TOF-MS/MS data of root, stem and leaf of *P. notoginseng*.


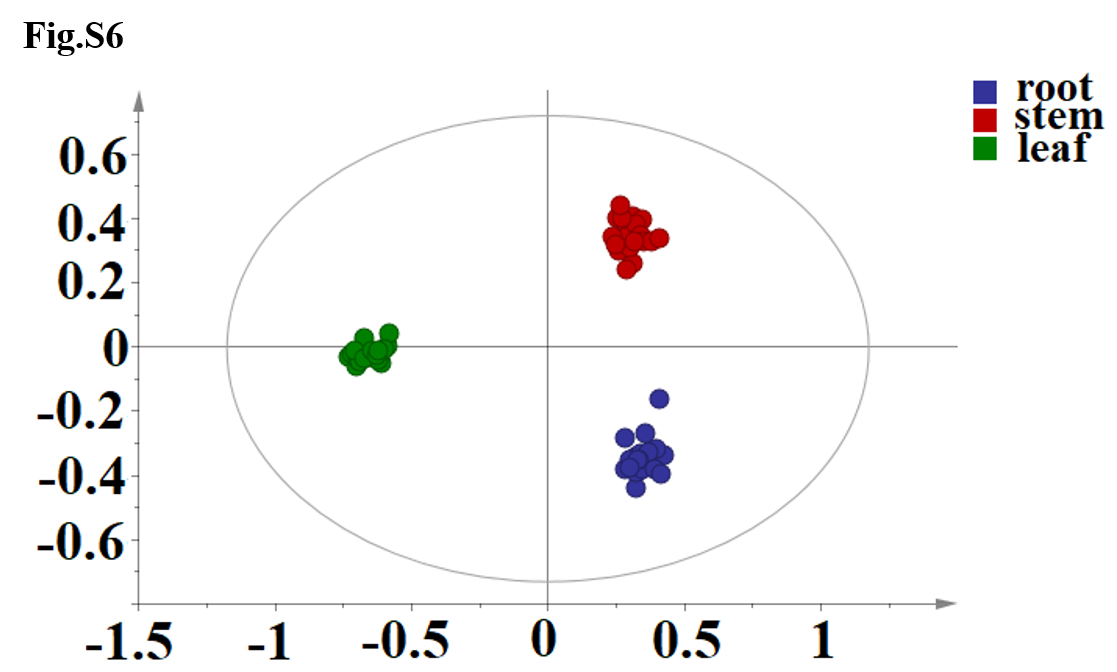


**Supplementary Fig. S6.** OPLS-DA scores plot of UHPLC-Q-TOF-MS/MS data of root, stem and leaf of *P. notoginseng*.


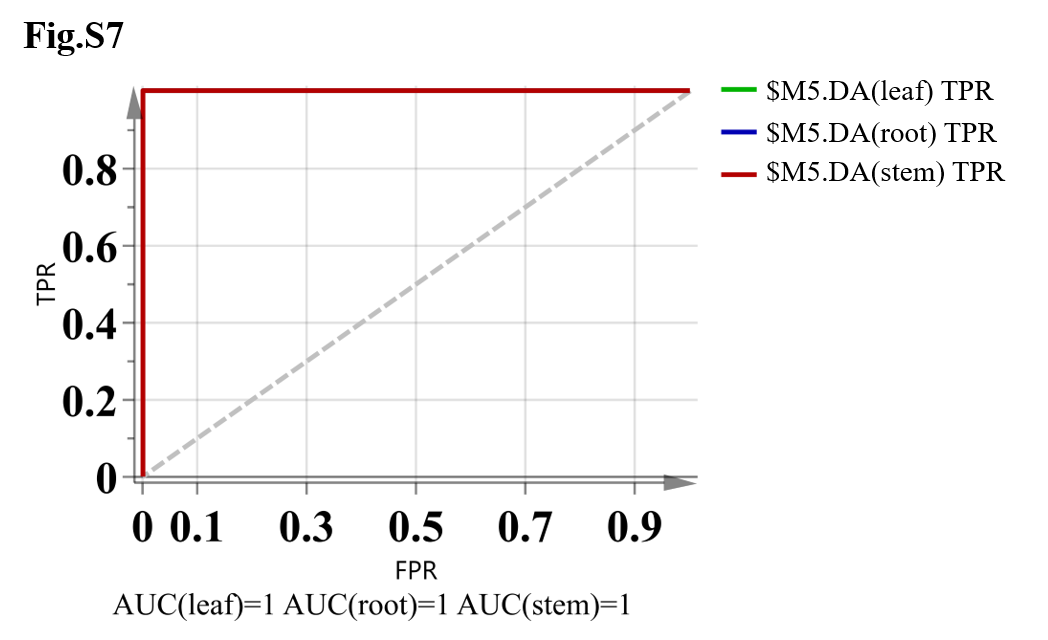


**Supplementary Fig. S7.** ROC analysis in prediction of root, stem and leaf of *P. notoginseng*.


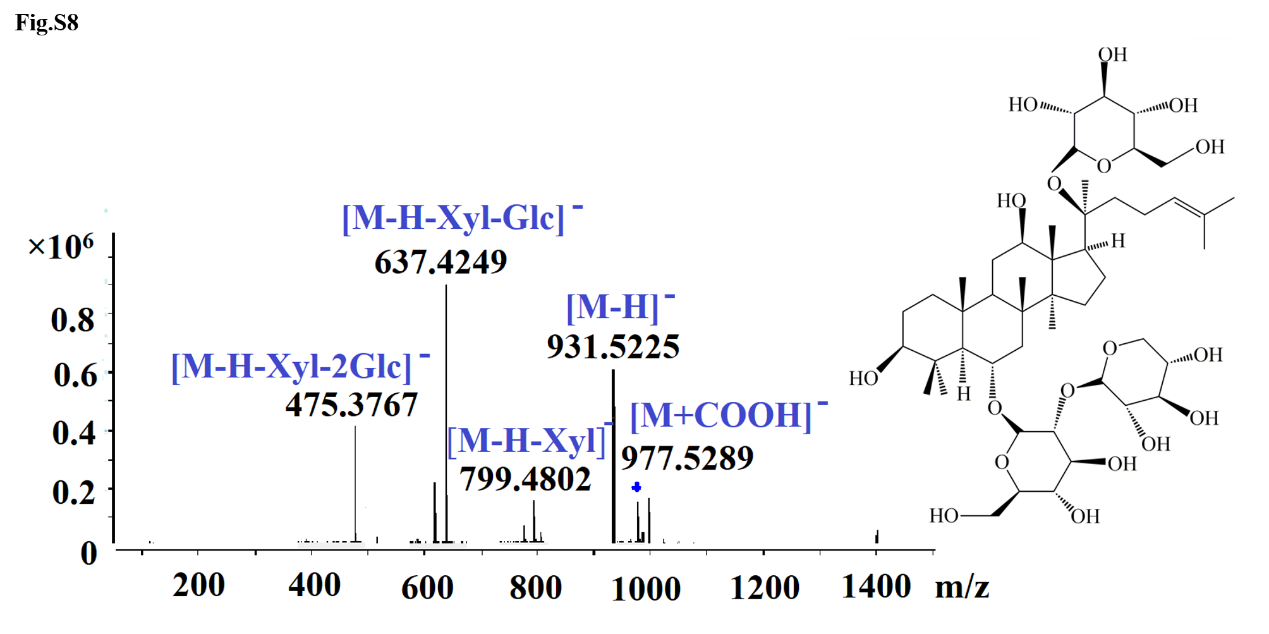


**Supplementary Fig. S8.** Targeted MS/MS of representative marker (notoginsenoside R1) contributing to the differentiation of different parts of *P. notoginseng*.


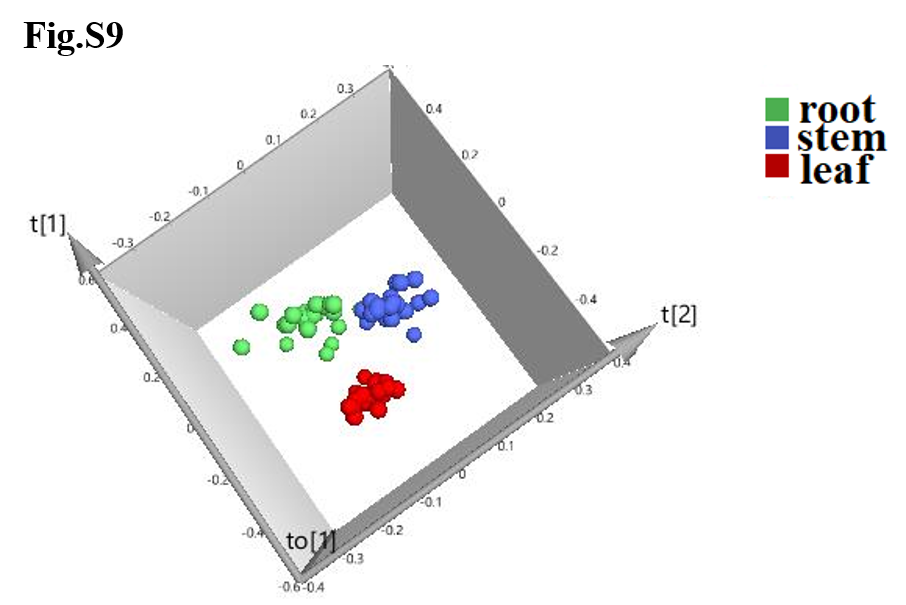


**Supplementary Fig. S9.** OPLS-DA scores plot of GC-MS data of root, stem and leaf of *P. notoginseng*.
